# Enantioselective synthesis of *gem*-diarylalkanes by transition metal-catalyzed asymmetric arylations (TMCAAr)

**DOI:** 10.1039/c7sc03404k

**Published:** 2017-11-08

**Authors:** Tao Jia, Peng Cao, Jian Liao

**Affiliations:** a Natural Products Research Center , Chengdu Institute of Biology , Chinese Academy of Sciences , Chengdu 610041 , People’s Republic of China . Email: jliao@cib.ac.cn; b College of Chemistry and Materials Science , Sichuan Normal University , Chengdu 610068 , People’s Republic of China . Email: caopeng@sicnu.edu.cn; c College of Chemical Engineering , Sichuan University , Chengdu 610065 , People’s Republic of China

## Abstract

Chiral *gem*(1,1)-diaryl containing tertiary or quaternary stereogenic centers are present in many natural products and important pharmacophores. While numerous catalytic asymmetric methods enable access to 1,1-diaryl motifs, transition metal-catalyzed asymmetric arylations (TMCAAr) are one of the most powerful methods to prepare enantiopure *gem*-diarylalkane compounds. The main methodology includes enantioselective 1,2- or 1,4-additions across C=O, C=N and C=C bonds by arylmetallic reagents; aryl cross-couplings of olefins, benzylic (pseudo)halides and aziridines; asymmetric aryl substitution reactions of allylic substrates; and isotopic benzylic C–H arylation.

## Abstract 2

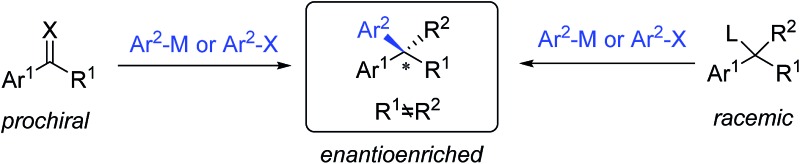
To date, enantiomerically enriched molecules containing *gem*(1,1)-diaryl containing tertiary or quaternary stereogenic centers have been readily accessed by transition metal-catalyzed enantioselective or stereoconvergent aryl transfer reactions.

## 1 Introduction

**Scheme 1:**
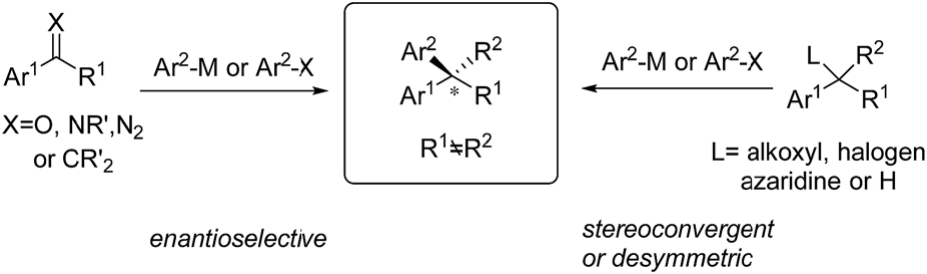
Conceptual strategies of TMCAAr.

Chiral *gem*(1,1)-diaryl containing tertiary or quaternary stereogenic centers are present in many natural products and important pharmacophores that possess distinct bioactivities, such as anticancer, antidepressant and antifungal properties and so on.^1^ In most cases, a single enantiomer (*R* or *S*) of *gem*-diarylalkanes is therapeutically effective and most medicinal molecules are approved in the optically pure form. Thus, the development of effective methods to access enantiomerically enriched diaryl structural motifs will play a significant role in both academic and industrial settings. Enantiomerically pure drugs or their precursors are usually produced by the chiral kinetic resolution technique. However, access to 1,1-diarylalkanes with a high level of optical purity using this technique is challenging because little differentiates the two aryl groups installed on the stereogenic center electronically and sterically. This issue can be solved by asymmetric synthetic methods through either stereospecific or enantioselective transformations. In the last few decades, an array of catalytic enantioselective approaches towards the construction of nonracemic *gem*-diaryl compounds have been developed, including asymmetric Friedel–Crafts reactions, asymmetric aryl transfer reactions (arylations), asymmetric hydrogenation of 1,1-diarylalkenes, asymmetric C–H functionalization of enantiotropic diarylalkanes and so on. Among them, transition metal-catalyzed asymmetric arylations (TMCAArs), which install an aryl group onto the benzylic position of substrates in an enantioselective or stereoconvergent manner, represent the most powerful method. In this field, development of new reactions, chiral ligand families and metal complexes has enabled the precise construction of various chiral diaryl motifs, including dibenzyl alkanes and alkenes, 1,1-diarylmethanols, 1,1-diarylmethylamines and so on. To the best of our knowledge, TMCAAr for the synthesis of *gem*-diaryl compounds includes nucleophilic 1,2- or 1,4-additions of arylmetallic reagents across C=O, C=N and C=C bonds; aryl cross-couplings to olefins, benzylic (pseudo)halides and aziridines; asymmetric aryl substitution reactions of allylic substrates; isotopic benzylic C–H arylation and so on (Scheme 1). These transformations feature a wide range of substrate scope, good functional group tolerance and the use of easily accessible feedstock chemicals. In contrast to conventional asymmetric methods, TMCAAr distinctively enable the assembly of both enantiomers through modulation of the reactants, instead of switching the absolute configuration of the chiral ligands.

**Fig. 1:**
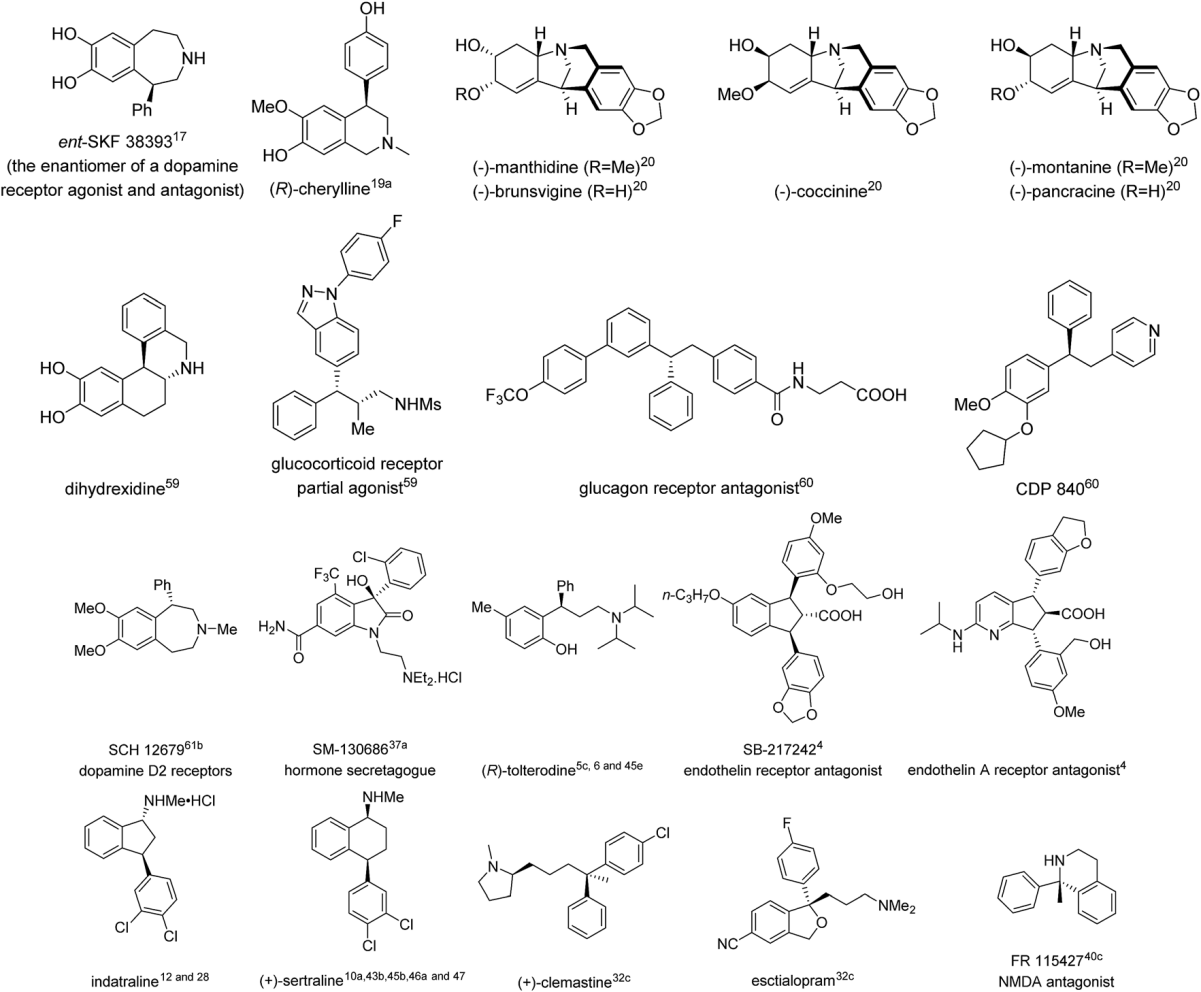
Representative natural products and bioactive compounds synthesised using TMCAAr.

To date, there have been many excellent reviews summarizing various asymmetric arylation strategies,^2^ some of which consist of most of the approaches towards 1,1-diarylmethanols and 1,1-diarylmethylamines. Hence, this review will focus on TMCAAr for the synthesis of chiral *gem*-diarylalkanes whose alkyl moieties contain at least two carbons. Additionally, transition metal-catalyzed intramolecular arylation reactions for the construction of *gem*-diaryl containing fused rings are not included herein. In this review, the literature is organized according to the reaction type as well as the category of prochiral substrates. Furthermore, the natural products as well as bioactive compounds prepared in this review are also listed in Fig. 1.

## 2 Asymmetric aryl addition to C=C, C=O and C=N bonds

**Scheme 2:**
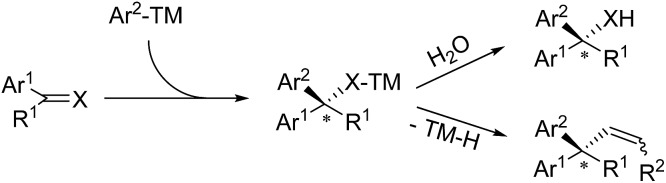
Conceptual strategies for conjugate or 1,2-arylation reactions.

Transition metal-catalyzed asymmetric aryl addition reactions to C=C, C=O and C=N bonds represent a highly efficient method to construct tertiary or quaternary stereogenic centers, concomitant with the formation of Csp^3^–Csp^2^ bonds. These transformations are frequently used to prepare important chiral *gem*-diaryl containing compounds from activated styrene and aryl-substituted carbonyl substrates. *Gem*-diaryl stereogenic centers are generated in the key step of aryl migratory insertion across the unsaturated C=C(O or N) bonds, followed by the hydrolysis or β-H elimination of the metal-binding intermediate (Scheme 2).

### 2.1 Conjugate additions to unsaturated carbonyl compounds

**Scheme 3:**
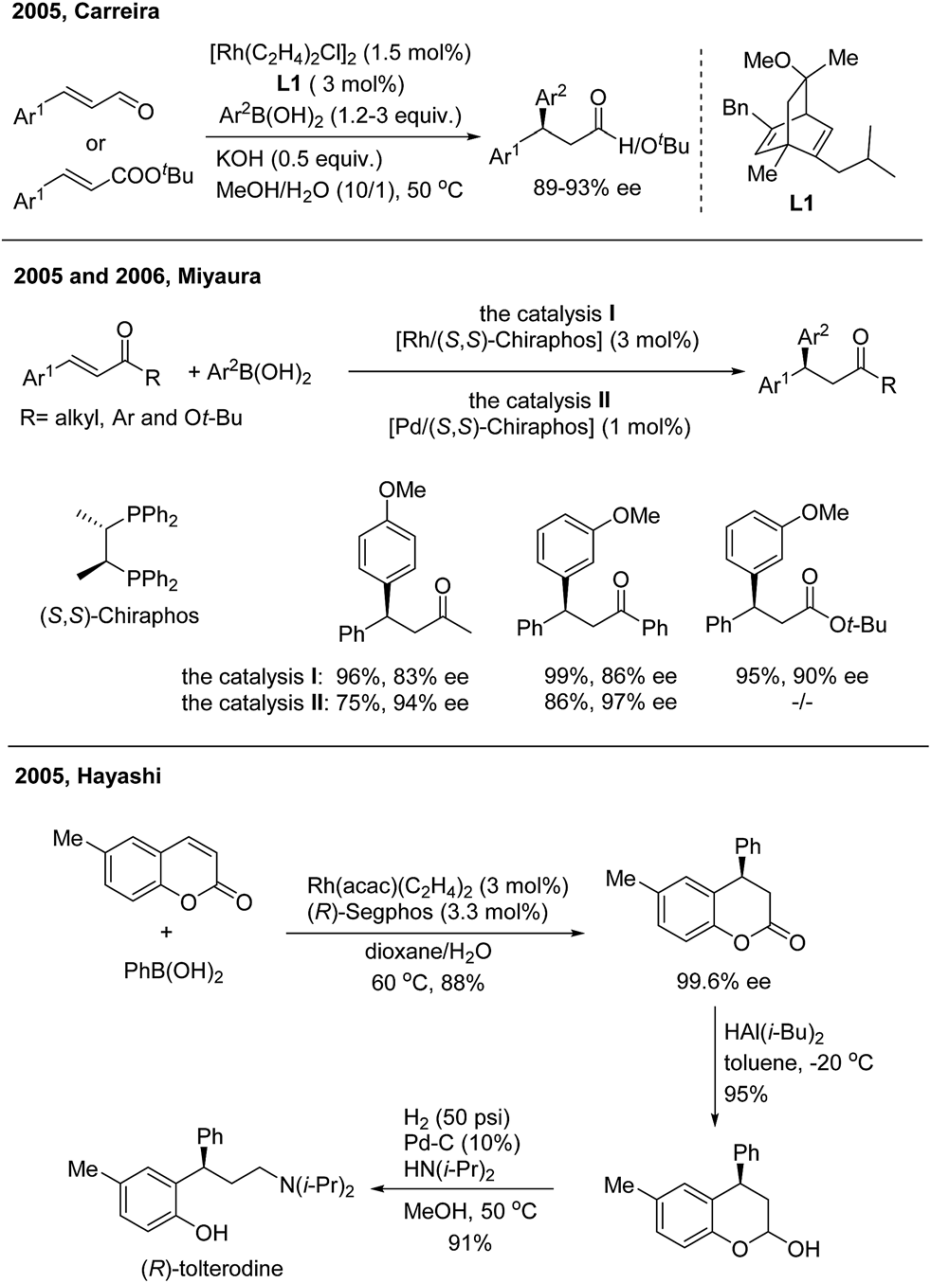
The enantioselective 1,4-addition of arylboronic acids to β-aryl substituted unsaturated carbonyl derivatives.

In 2005, Carreira successfully realized Rh(i)-catalyzed highly enantioselective 1,4-addition of arylboronic acids to β-aryl substituted unsaturated carbonyl derivatives using a carvone-derived chiral diene ligand (**L1**)^3^ (Scheme 3, top). Enantioenriched 3,3-diarylpropanals and *tert*-butyl 3,3-diarylpropanoates were afforded with 89–93% ee. Miyaura found that both Rh(i)^4^ and Pd(ii)^5^ complexes with a (*S*,*S*)-chiraphos ligand are competent catalysts for TMCAAr of β-aryl-α,β-unsaturated ketones and esters (Scheme 3, middle). However, attempts to use indenone as the Michael acceptor gave only 20% yield of a nearly racemic product, the reason for which is yet unsolved. Hayashi found that coumarins undergo 1,4-arylation using a Rh/(*R*)-Segphos catalyst to provide enantiomerically pure 4-arylchroman-2-ones. The product, (*R*)-6-methyl-4-phenylchroman-2-one, was readily converted in two steps into (*R*)-tolterodine, an important urological drug^6^ (Scheme 3, bottom). For the 1,4-arylation of chalcones, Liao and coworkers demonstrated that a Rh(i) complex of sulfoxide–phosphine was an appropriate catalyst to afford chiral 1,3,3-triarylpropan-1-ones with up to 98% ee.^7^

**Scheme 4:**
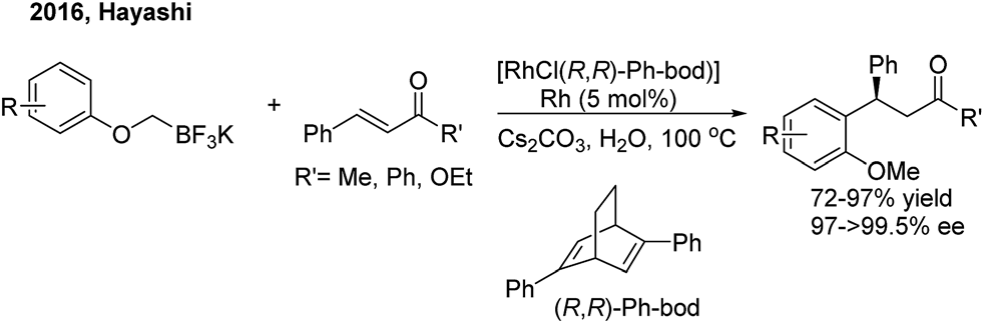
Rh-catalyzed migration/arylation of α,β-unsaturated carbonyl compounds.

In 2016, Hayashi employed the 1,4-Rh migration/arylation strategy to realize conjugate addition of potassium aryloxymethyltrifluoroborates to α,β-unsaturated carbonyl compounds in the presence of a chiral diene–rhodium catalyst^8^ (Scheme 4). The desired β,β-diaryl ketones or esters were afforded in high yields with excellent enantioselectivities.

**Scheme 5:**
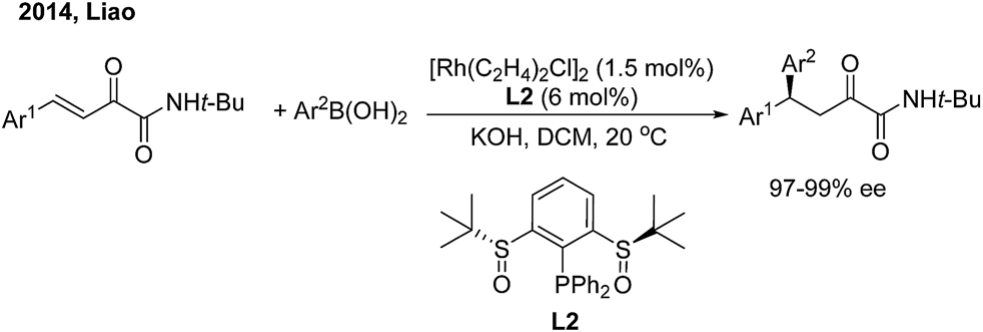
Rh-catalyzed enantioselective 1,4-additon of β,γ-unsaturated-α-ketoamides.

While chiral olefins or phosphines enable control of the 1,4-regioselectivity of α,β-unsaturated aldehyde/ketone/ester substrates, the conjugate arylation of β,γ-unsaturated α-keto carbonyl compounds is difficult to realize using these ligands, which only promote 1,2-addition.^9^ In 2014, Liao and co-workers^10^ demonstrated a highly regio- and enantioselective Rh-catalyzed 1,4-addition of arylboronic acids to β,γ-unsaturated α-keto carbonyl derivatives using a novel chiral sulfoxide–phosphine ligand (**L2**) (Scheme 5). Nonracemic γ,γ-diaryl, α-keto amides and esters were produced. The method was applied in the concise syntheses of sertraline and tetrahydroquinoline-2-carboxylamide.

**Scheme 6:**
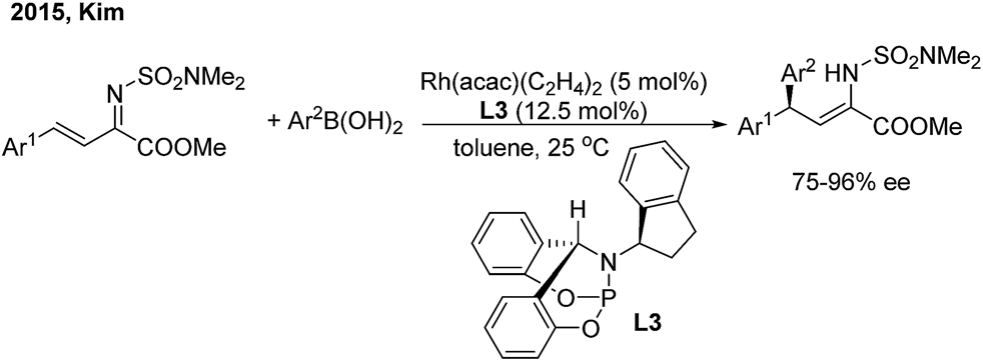
Rh-catalyzed asymmetric 1,4-addition of arylboronic acids to α,β-unsaturated *N*,*N*-dimethylsulfamoyl imino esters.

In 2015, Kim and co-workers^11^ reported an elegant Rh-catalyzed asymmetric 1,4-addition of arylboronic acids to α,β-unsaturated *N*,*N*-dimethylsulfamoyl imino esters with a new bicyclic bridgehead phosphoramidite ligand (**L3**). Chiral (*Z*)-γ,γ-diaryl-α,β-dehydroamino esters were afforded with excellent yields and enantioselectivities (75–96% ee) (Scheme 6).

**Scheme 7:**
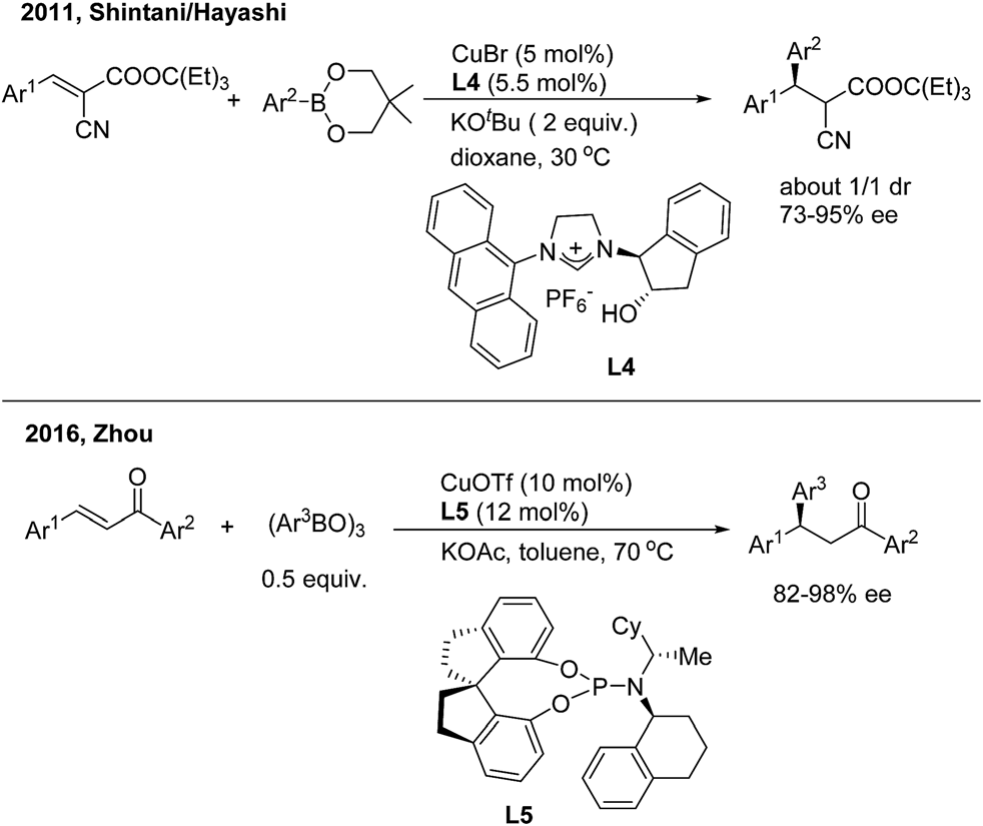
Cu-catalyzed enantioselective 1,4-addition of α,β-unsaturated carbonyl derivatives.

Asymmetric 1,4-addition of organoboronates to alkylidene cyanoacetates by copper catalysis was first demonstrated by Shintani/Hayashi^12^ using a chiral *N*-heterocyclic carbene ligand (**L4**) (Scheme 7, top). The transformation releases optically active 2-cyano-3,3-diaryl propanoates as a mixture of diastereomers (1 : 1). The author conducted a series of stoichiometric reactions and indicated that only copper(i) mediated the catalytic cycle that consists of transmetalation/insertion/ligand exchange. Zhou and coworkers^13^ recently found that the chiral copper complex of phosphoramidite (**L5**) efficiently promoted the enantioselective 1,4-addition of chalcones with arylboroxines and a direct 1,4-insertion mechanism was proposed and supported by DFT calculations and natural-abundance ^13^C KIE experiments (Scheme 7, bottom).

### 2.2 Conjugate additions to nitro or sulfonyl olefins

**Scheme 8:**
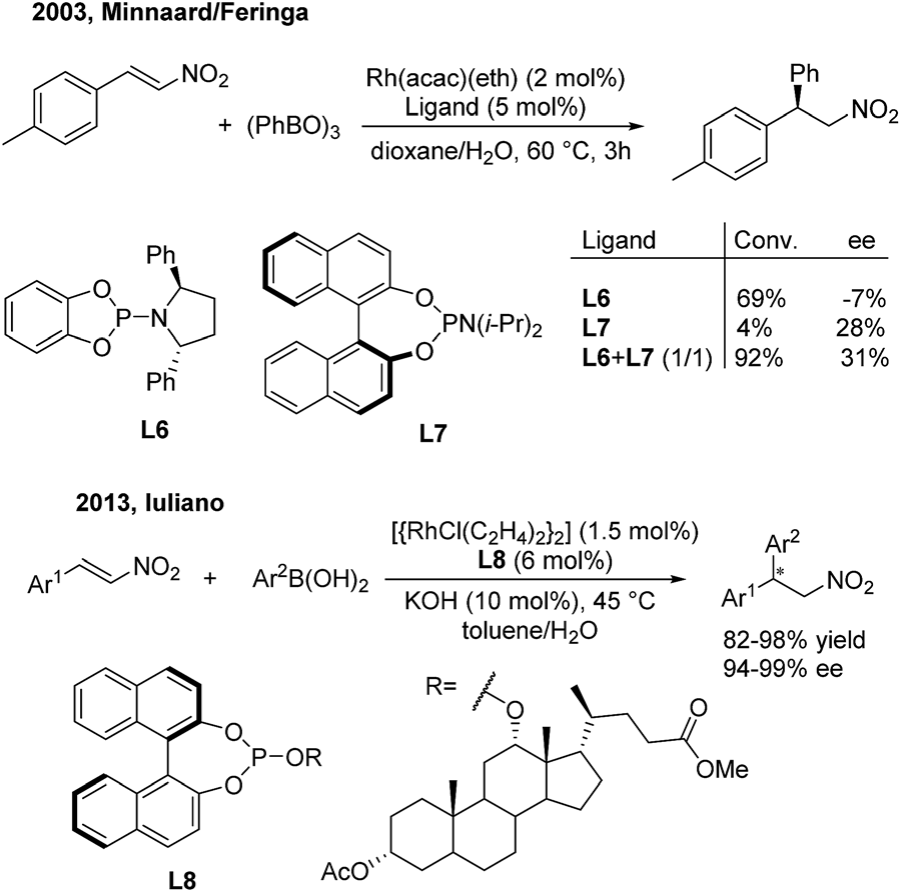
Rh(i)-catalyzed conjugate addition of arylboron reagents to β-aryl nitroethylenes using chiral phosphoramidite and phosphite ligands.

In 2003, Minnaard/Feringa^14^ reported the first rhodium-catalyzed asymmetric addition of triphenyl boroxine to β-aryl nitroethylenes using chiral phosphoramidite ligands. Chiral 2,2-diaryl nitroethanes were afforded in excellent conversion and modest enantioselectivities. In this reaction, **L6** could give 69% conversion but a low ee value (–7%), while the more sterically hindered **L7** gave 28% ee but a low conversion (4%) (Scheme 8, top). Interestingly, the combination of **L6** and **L7** in a 1 : 1 ratio could improve the conversion (92%) as well as the enantioselectivity (31%). In 2013, Iuliano^15^ demonstrated that the deoxycholic acid-derived mono-Phos (**L8**) significantly improved the enantioselectivities (94–99%) as well as the yields (82–98%) of the desired products (Scheme 8, bottom).

**Scheme 9:**
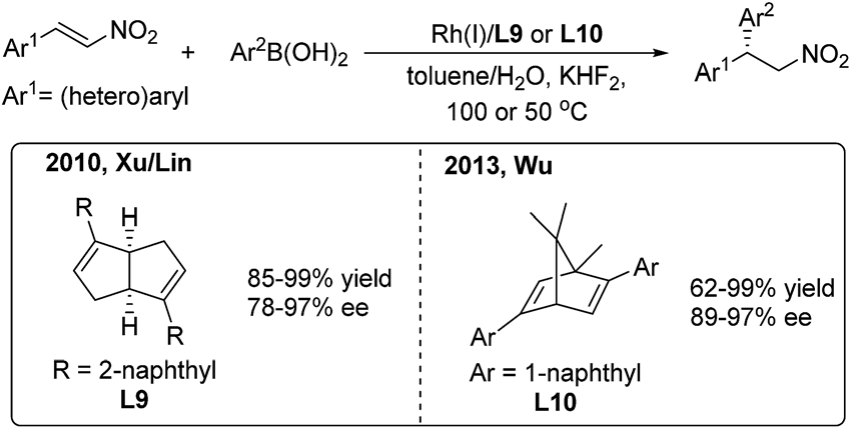
Rh(i)-catalyzed conjugate addition of arylboronic acids to β-aryl nitroethylenes using chiral diene ligands.

In 2010, Xu/Lin^16^ reported a highly enantioselective addition of organoboronic acids to nitroalkenes using a rhodium/chiral diene catalyst (Scheme 9, left). Enantioenriched 2,2-diaryl nitroalkanes were obtained with moderate to good enantioselectivities (78–97%) induced by the chiral [3.3.0]-diene ligand (**L9**). In 2013, Wu and coworkers^17^ used a chiral [2.2.1]-diene ligand (**L10**) in the arylation of nitroalkenes with high enantioselectivities (89–97%) (Scheme 9, right). The catalyst loading of the model reaction can be reduced to 0.1 mol%. Recently, Wu found that the amide-containing *C*_1_-symmetric [2.2.2]-diene ligand can promote the enantioselective reaction at room temperature.^18^

**Scheme 10:**
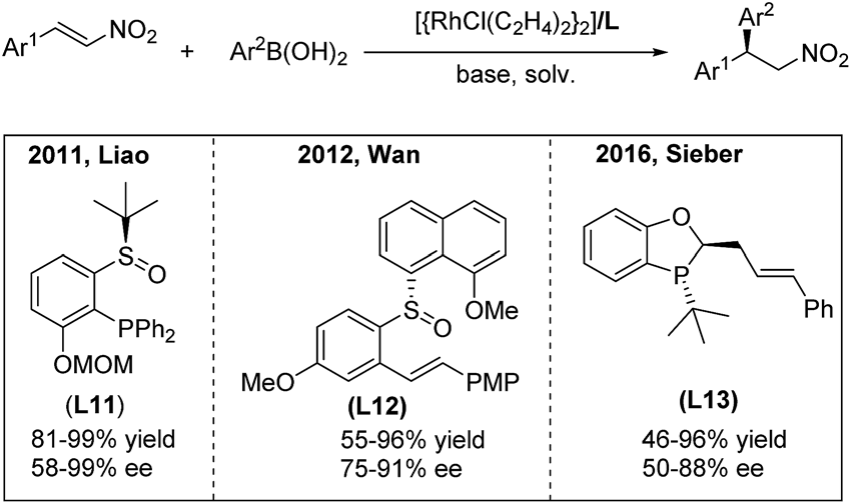
Rh(i)-catalyzed conjugate addition of arylboronic acids to β-aryl nitroethylenes using chiral hybrid ligands.

In 2011, the highly efficient rhodium-catalyzed enantioselective addition of arylboronic acids to β-aryl and β-indolyl nitroalkenes was developed by the Liao group using the chiral sulfoxide–phosphine (SOP) ligand **L11**^19^ (Scheme 10, left). Moreover, the utility of this method was documented by Fan in the synthesis of montanine-type amaryllidaceae alkaloids.^20^ In 2012, Wan and co-workers^21^ reported a rhodium-catalyzed asymmetric addition of arylboronic acids to nitroalkenes using the chiral sulfoxide–olefin ligand **L12** (Scheme 10, middle). They successfully enlarged the scope of the reaction to aryl, alkyl and heteroaryl nitroalkenes in one catalytic system. Recently, a P-chiral phosphine–olefin hybrid ligand **L13** has been demonstrated by Sieber to efficiently promote this reaction^22^ (Scheme 10, right).

**Scheme 11:**
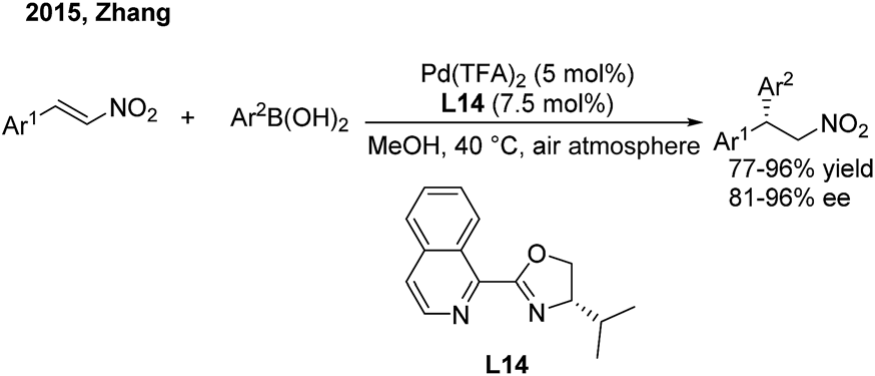
Pd(ii)-catalyzed conjugate addition of arylboronic acids to β-aryl nitroethylenes using chiral IsoQuinox ligands.

Recently, Zhang and coworkers^23^ devoted themselves to developing a cheap and robust palladium catalysis system for conjugate aryl addition to nitroethylenes. When using iPr-IsoQuinox (**L14**) as a chiral ligand, enantioenriched 2,2-diaryl nitroalkanes can be produced in high yields and good enantioselectivities in air (Scheme 11).

**Scheme 12:**
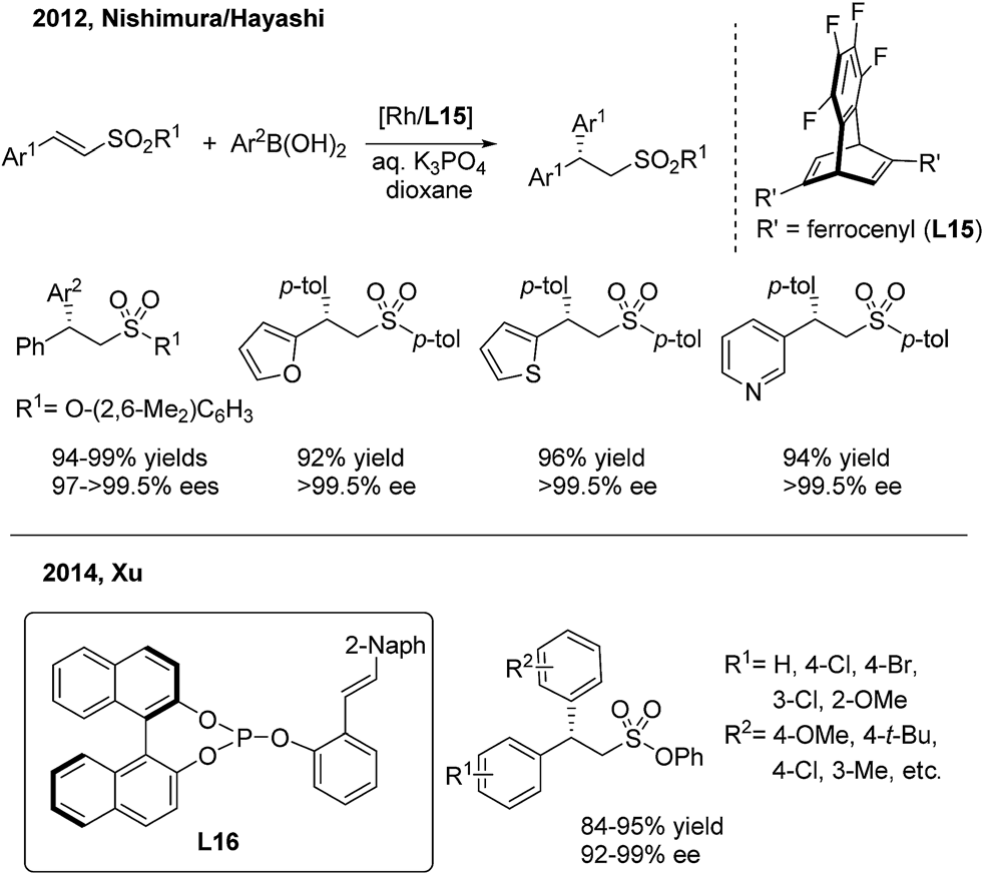
Rh(i)-catalyzed conjugate aryl addition to α,β-unsaturated sulfonyl compounds.

In contrast to the extensive studies on conjugate arylations of nitroalkene substrates, successful conjugate additions of sulfonyl olefins have rarely been reported.^24^ In 2012, Nishimura and Hayashi disclosed an elegant enantioselective addition of arylboronic acids to α,β-unsaturated sulfonyl compounds with a high enantioselectivity (97 to >99.5% ee)^25^ (Scheme 12, top). They demonstrated that the use of a diene ligand (**L15**) induces the protonation of the alkylrhodium intermediate faster than the β-H elimination process, thus selectively forming the addition product instead of the substitution product. Later on, Xu employed the chiral phosphine–olefin ligand (**L16**) in the same asymmetric reaction to achieve generally high yields and ee values^26^ (Scheme 12, bottom).

### 2.3 Asymmetric Heck-type addition to unactivated styrenes

**Scheme 13:**
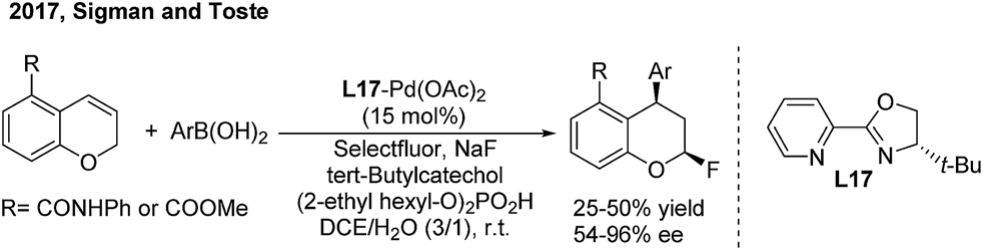
Pd-catalyzed enantioselective 1,3-arylfluorination of chromenes.

Recently, Sigman and Toste disclosed a palladium-catalyzed 1,3-regio and *syn*-diastereoselective arylfluorination of chromenes with arylboronic acids and selectfluor.^27^ With (*S*)-4-*tert*-butyl-2-(2-pyridyl)oxazoline (**L17**) as the chiral ligand, a wide spectrum of enantioenriched 2-fluoro-4-phenylchromanes were produced with up to 96% ee, albeit in moderate yields (Scheme 13). Meanwhile, an oxidative Heck-like mechanism was proposed based on the experimental studies in combination with computational and statistical analysis tools.

**Scheme 14:**
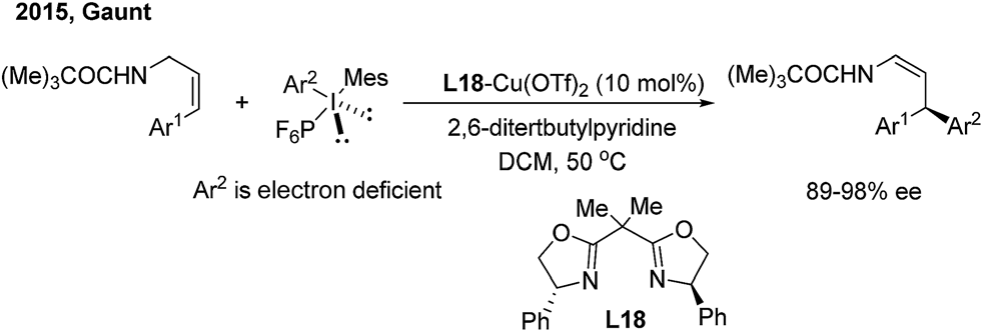
Cu/BOX-catalyzed enantioselective electrophilic arylation of allylic amides.

In contrast to nucleophilic arylation, Gaunt recently reported a novel copper/bisoxazoline (**L18**)-catalyzed electrophilic arylation of allylic amides^28^ (Scheme 14). The protocol enables the asymmetric transfer of the electron-poor aryl group of diaryliodonium salts to the γ position of cinnamyl amides and provides chiral β,β-diaryl enamides with a high level of optical purity.

### 2.4 1,2-Addition to arylketone and arylketimine derivatives

**Scheme 15:**
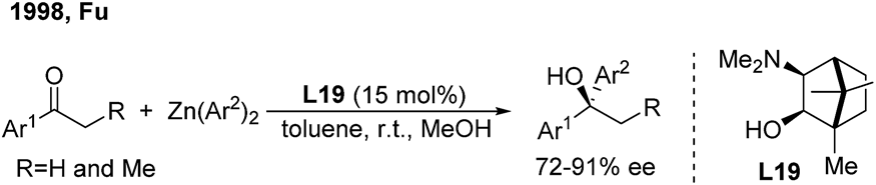
The first catalytic asymmetric addition of organometallic reagents to ketones.

For ketone arylations, Fu reported the first enantioselective 1,2-addition of Ph_2_Zn to unactivated ketones catalyzed by 3-*exo*-(dimethylamino)isoborneol (**L19**)^29^ (Scheme 15). Although both aryl-alkyl and dialkyl ketones are reactive in the presence of MeOH, aryl-alkyl ketones gave better enantioselectivities (72–91%). Later on, Walsh and Yus/Ramón independently demonstrated that the easily accessible chiral isoborneolsulfonamide and camphorsulfonamide are good ligands.^30^ The catalytic system consisting of a combination of chiral diol ligands and Ti(O^i^Pr)_4_ also promoted the enantioselective addition of Ph_3_Al, ArTi(O^i^Pr)_3_ and ArMgBr to ketones, producing chiral diaryl alkyl carbinols.^31^

**Scheme 16:**
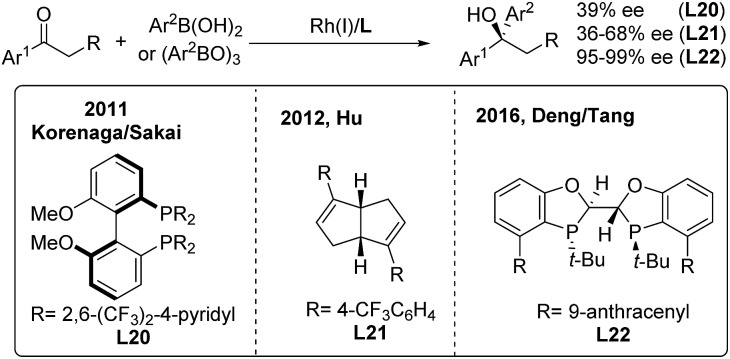
Rh-catalyzed enantioselective 1,2-addition of α-aryl ketones.

While arylboronic acids or derivatives are stable and frequently used in transition-metal catalyzed arylation reactions, their enantioselective additions to unactivated ketones are limited,^32^ probably due to the lack of effective chiral ligands. In 2011, Sakai/Korenaga32*a* discovered that electron-poor 2,6-bis(trifluoromethyl)-4-pyridyl (BFPy) phosphanes enable the acceleration of the Rh-catalyzed 1,2-addition of arylboronic acids to ketones. Accordingly, the enantioselective variant was obtained using BFPy derived biphep (**L20**) as the chiral ligand, albeit with only 39% ee (Scheme 16, left). Later on, a chiral diene ligand (**L21**) was demonstrated to promote the addition of arylborons to cyclic or acyclic arylketones with up to 68% ee32*b* (Scheme 16, middle). Recently, Deng and Tang32*c* reported a highly enantioselective addition of arylboroxines to simple aryl ketones catalyzed by the Rh/**L22** complex, which produced a range of chiral diaryl alkyl carbinols with excellent ee (95–99%) (Scheme 16, right). The utility of this method was illustrated by the concise synthesis of the antidepressant drug escitalopram as well as the (+)-clemastine intermediate.

**Scheme 17:**
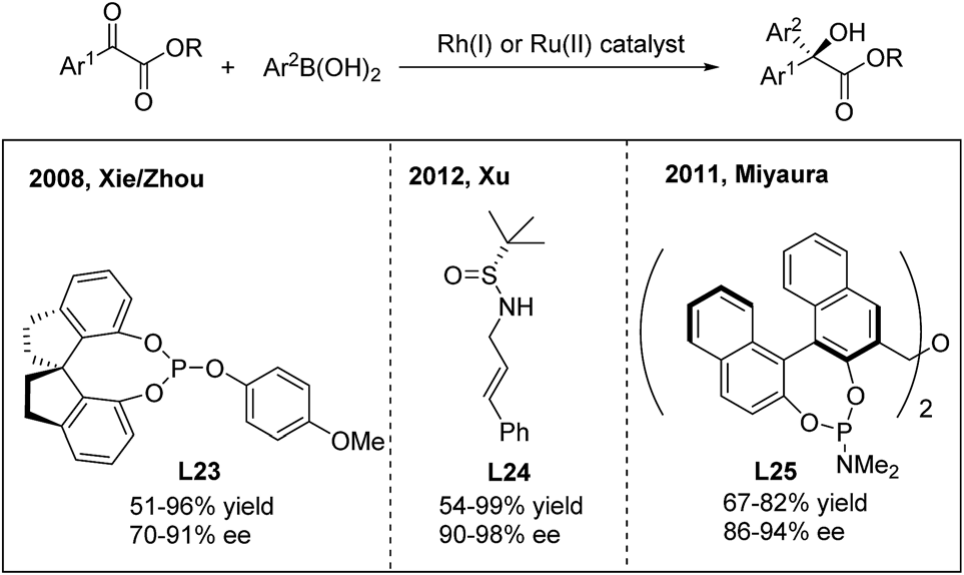
Catalytic asymmetric 1,2-addition of arylboronic acids to α-ketoesters.

For the 1,2-arylation of activated ketones, Xie and Zhou developed the first highly enantioselective addition of arylboronic acids to α-ketoesters using a chiral Rh(i)-spirophosphite (**L23**) catalyst33*a* (Scheme 17). The method allows the synthesis of α-hydroxy-α-diaryl acetates with moderate to high ee (70–91%). A few years later, Xu and coworkers employed a simple *N*-(sulfinyl)cinnamylamine ligand (**L24**) in the arylation of α-ketoesters and α-diketones.33*b* Highly enantiopure α-hydroxy-α-diaryl acetates were afforded. In addition to rhodium catalysis, a ruthenium complex generated from [RuCl_2_(*p*-cymene)]_2_ and (*R*,*R*)-Me-BIPAM (**L25**) could also promote the asymmetric addition of arylboronic acids to α-ketoesters with high enantioselectivities.33*c*

**Scheme 18:**
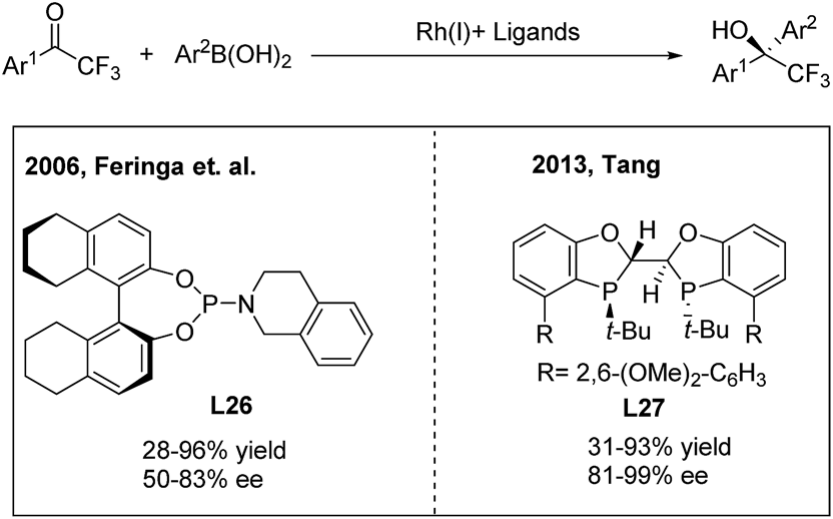
Rh-catalyzed asymmetric 1,2-addition of arylboronic acids to 2,2,2-trifluoroacetophenones.

Due to the unique biological activities of fluorinated compounds, many scientists focused on the development of catalytic asymmetric methods for the synthesis of α-chiral CF_3_-containing compounds. However, enantioselective synthesis of diaryl trifluoroethanes through TMCAAr has rarely been reported.^34^ In 2006, Vries, Feringa and Minnaard34*a* reported the first asymmetric approach towards 2-hydroxy-2,2-diaryl trifluoroethanes through the rhodium(i)/phosphoramidite (**L26**) catalysed 1,2-addition of arylboronic acids to 2,2,2-trifluoroacetophenones (Scheme 18, left). In 2010, Iuliano and coworkers34*b* found that optically active 2-hydroxy-2,2-diaryl trifluoroethanes could also be produced using a deoxycholic acid derived monophosphite as the chiral ligand, albeit with moderate enantioselectivities. Recently, Tang34*c* demonstrated that a new *C*_2_-symmetrical chiral bisphosphorus ligand (**L27**) was highly effective in the Rh-catalyzed arylation of trifluoroacetophenones (Scheme 18, right).

**Scheme 19:**
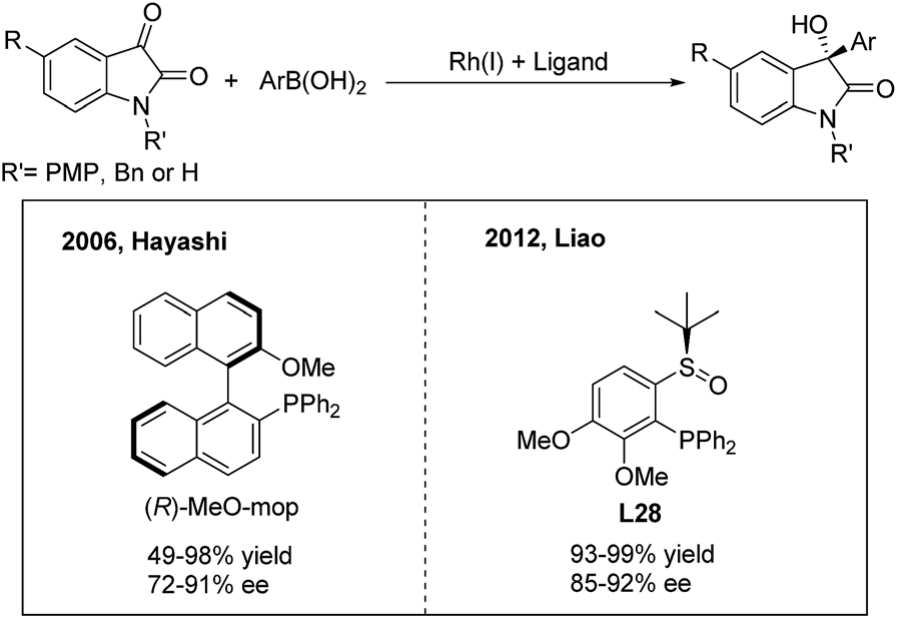
Catalytic asymmetric 1,2-addition of arylboronic acids to isatins.

3-Hydroxy-3-aryl-2-oxindoles are important biologically active candidates in recent pharmaceutical studies. The Rh/Ir(i),^35^ Pd(ii),^36^ Cu(i)^37^ and Ru(ii)^38^-catalyzed enantioselective additions of arylboronic acids or esters to isatins and derivatives provide efficient methods for the synthesis of these compounds. In 2006, Shintani and Hayashi35*a* reported the first rhodium-catalyzed asymmetric addition of arylboronic acids to isatins using (*R*)-MeO-mop as a chiral ligand. A variety of optically active 3-hydroxy-3-aryl-2-oxindoles were afforded in good to excellent yields (49–98%) with high enantioselectivities (72–91%) (Scheme 19, left). Meanwhile, Vries, Feringa and Minnaard35*b* examined a chiral phosphoramidite in the Rh(i)-catalyzed arylation of NH isatin but obtained a poor enantioselectivity (55%). Liao and co-workers35*c* demonstrated that the chiral sulfoxide–phosphine (SOP) ligand **L28** is also compatible with the NH isatin arylation process and gives an improved efficiency (Scheme 19, right).

**Scheme 20:**
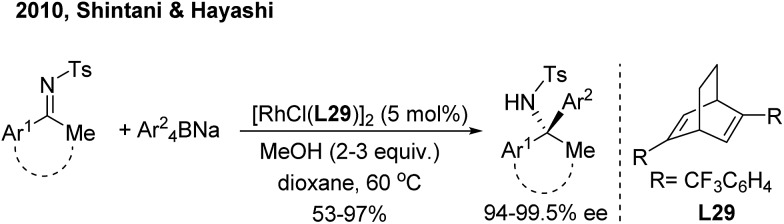
Rh/diene-catalyzed enantioselective 1,2-arylation of ketimines.

For ketimine arylation, Hayashi/Shintani^39^ pioneered the Rh-catalyzed asymmetric arylation of *N*-tosyl ketimines with sodium tetraarylborates by employing a chiral diene ligand (**L29**) (Scheme 20). The method is practically useful for the synthesis of chiral arylethanone-, indanone- and tetralone-derived amines.

**Scheme 21:**
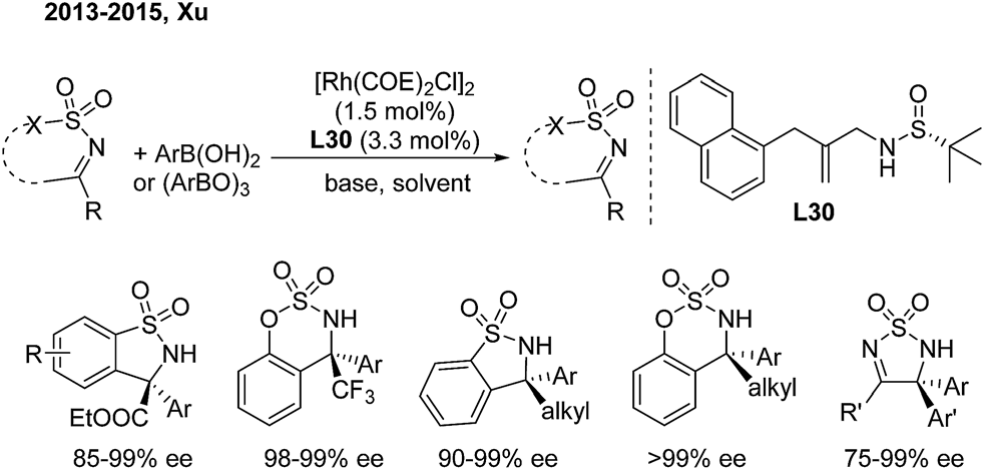
Rh/sulfur–olefin-catalyzed enantioselective 1,2-addition of ketimines.

Benzosultams containing a chiral α-amino acid unit and benzosulfamidates containing a CF_3_ group are attractive to organic and medicinal chemists. In 2013, Xu and coworkers developed a rhodium-catalyzed asymmetric addition of arylboronic acids to CF_3_- or alkoxycarbonyl-substituted cyclic ketimines.40*a* In this reaction, they utilized a chiral sulfur–olefin ligand (**L30**) which they developed themselves to provide such molecules in high yields with excellent enantioselectivities (Scheme 21). The analogous alkyl-substituted cyclic *N*-sulfonyl ketimines can also produce enantioenriched α-arylalkyl-substituted benzosulfamidates and benzosultams with excellent ee.40b,c These adducts allow for further transformation to versatile chiral α-diaryl alkylamines and some bioactive analogues.

**Scheme 22:**
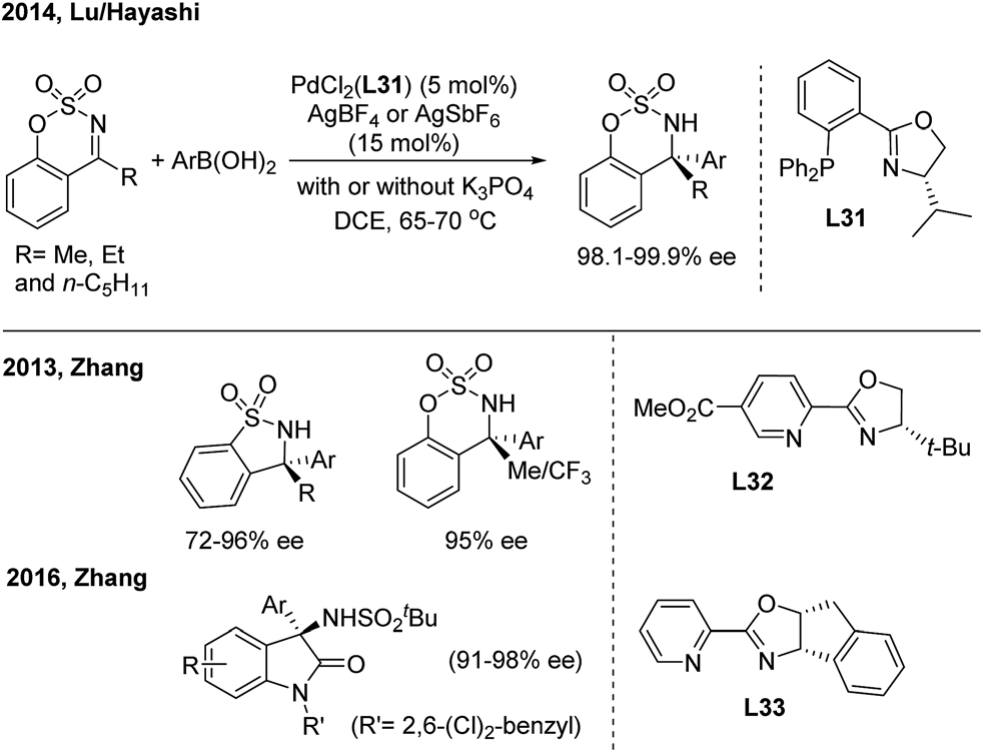
Pd-catalyzed 1,2-addition of arylboronic acids to cyclic ketimines.

Pd-catalyzed enantioselective additions of arylboronic acids to cyclic *N*-sulfonyl ketimines were disclosed by Zhang41*a* and Lu/Hayashi^42^ using chiral pyridine-oxazoline (**L32**) and phosphine-oxazoline (**L31**) ligands, respectively (Scheme 22). Analogously, the enantioselective 1,2-addition of arylboronic acids to 3-ketimino oxindoles, by the Zhang group,41*b* was catalysed by a Pd(ii)/**L33** complex and enables the synthesis of enantioenriched 3-amino-3-aryl-2-oxindoles with high ee. Zhang also demonstrated the first Ni(ii)-catalyzed asymmetric addition of arylboronic acids to cyclic imines using a tropos phosphine-oxazoline biphenyl ligand.41*c*

## 3 Asymmetric allylic arylation (AAAr) reactions

**Scheme 23:**
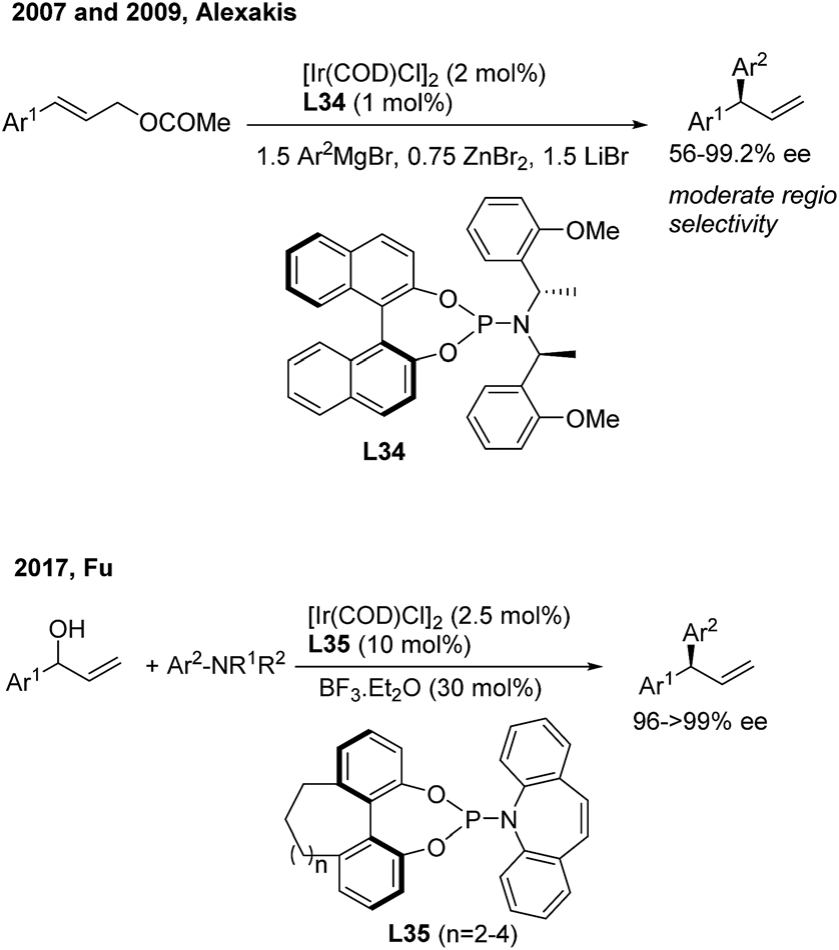
Ir(i)-catalyzed AAAr using chiral phosphoramidite ligands.

Asymmetric allylic arylation (AAAr) reactions of cinnamyl electrophiles are one of the most important strategies to access chiral 1,1-diarylpropene molecules. Although the transfer of aryl groups to γ-aryl substituted substrates resulted mainly in the achiral α product with palladium catalysis, the γ-regioselectivity is facile for iridium and copper catalysis. In 2007, Alexakis^43^ reported the first AAAr of arylzinc reagents to cinnamyl carbonates catalyzed by chiral Ir(i)/**L34** complexes, which afforded the γ product with a high enantioselectivity but moderate γ-regioselectivity (Scheme 23, top). Recently, Fu^44^ realized the Ir/**L35**-catalyzed enantioselective arylation of racemic secondary allylic alcohols with aniline derivatives using BF_3_·Et_2_O (30 mol%) as the promoter. The formal S_N_2-substituted products, *gem*-diarylpropenes, were obtained with excellent ee (Scheme 23, bottom).

**Scheme 24:**
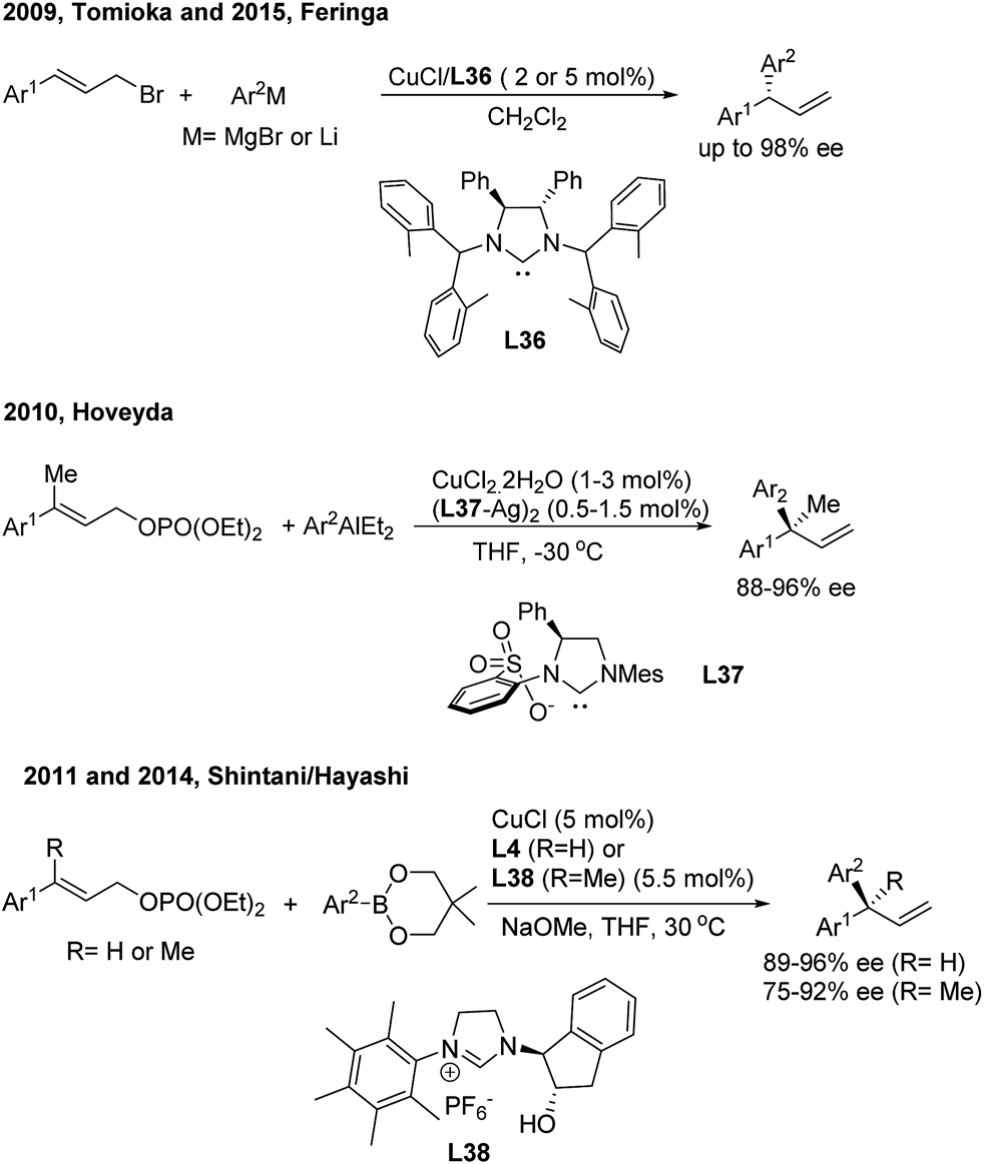
Cu-catalyzed AAAr using chiral NHC–carbene ligands.

In the field of Cu(i)-catalyzed AAAr,^45^ chiral *N*-heterocyclic carbenes (**L36**, **L37**, **L4** and **L38**) displayed remarkably high γ-regioselectivity as well as excellent enantioselectivity (Scheme 24). In these transformations, an array of arylmetallic (*i.e.* Mg, Li, Al and B) reagents can couple with cinnamyl bromides or carbonates to construct tertiary and quaternary *gem*-diarylmethine stereogenic centres.

## 4 Asymmetric aryl cross-coupling to benzyl C–X bonds

### 4.1 Enantioconvergent cross-coupling reactions of racemic benzylic substrates

**Scheme 25:**
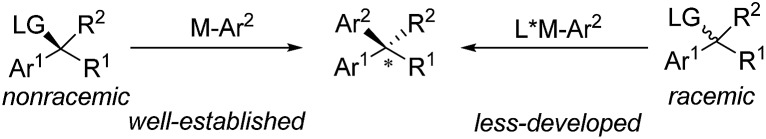
The conceptual strategies for asymmetric cross-coupling of benzyl electrophiles and arylmetallic reagents.

Transition metal-catalyzed stereospecific aryl cross-couplings allow for the transformation of secondary enantioenriched benzylic electrophiles or nucleophiles to 1,1-diarylalkane compounds. However, the catalytic enantioselective transformations of racemic benzylic compounds to enantiomerically enriched products still remain limited^46^ (Scheme 25).

**Scheme 26:**
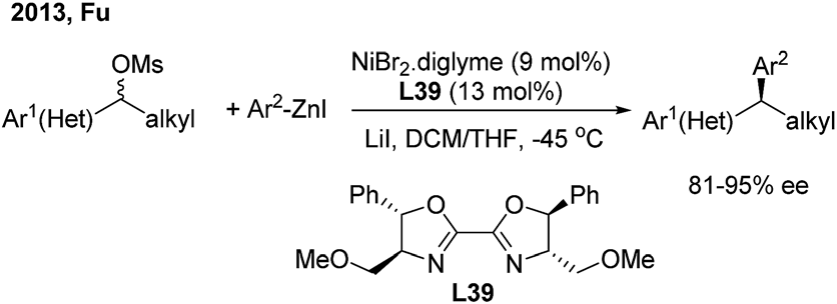
Ni/BOX-catalyzed enantioconvergent Negishi reactions of racemic benzylic electrophiles.

**Scheme 27:**
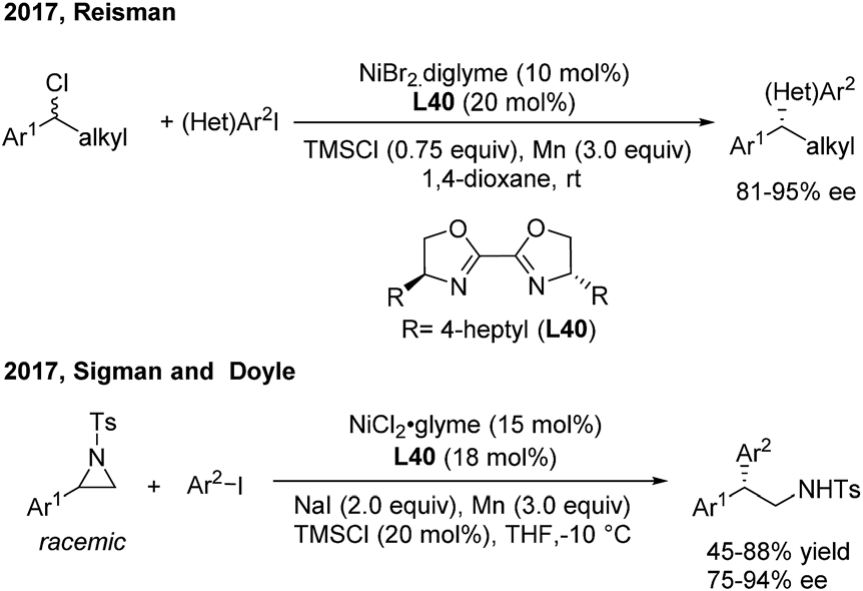
Ni/BiOx-catalyzed stereoconvergent reductive cross-coupling of racemic styrenyl aziridines and aryl iodides.

In 2013, Fu46*a* developed the first successful enantioconvergent Negishi reactions of racemic benzylic mesylates with arylzinc reagents (Scheme 26). A broad range of 1,1-diarylalkanes with a high level of optical purity (81–95% ee) were produced when using a chiral nickel(ii)/**L39** catalyst. The method was applied to a gram-scale synthesis of (*S*)-sertraline tetralone from the available racemic 4-hydroxy-4-phenylbutanoate. Other efforts to attempt the enantioconvergent arylation of racemic benzylic chloride or trifluoroborate, also by Ni(ii)/bis(oxazoline) catalysis, revealed the moderate stereoselectivity. In 2017, Reisman^47^ disclosed an elegant enantioselective Ni-catalyzed reductive cross-coupling between racemic secondary benzylic chlorides and (hetero)aryl iodides with 4-heptyl substituted bioxazoline (**L40**) as the chiral ligand (Scheme 27, top). In particular, 5-iodo-2-substituted pyridines were quite reactive under standard conditions and a wide range of 1,1-diarylalkanes were prepared with a generally high enantiopurity.

The catalytic asymmetric α-arylation of styrenyl aziridines is one of the most important methods to access nonracemic 2,2-diarylethylamine derivatives. However, successful cross-coupling reactions rely on the stereospecific transformation of enantiomerically enriched aziridines. Recently, Sigman and Doyle developed an elegant Ni-catalyzed stereoconvergent reductive cross-coupling of racemic *N*-Ts aziridines and aryl iodides with Mn(0) as the reductant (Scheme 27, bottom). Intrigued by the discovery that enantiopure aziridine produces the corresponding amine as the racemate, they examined chiral amine- and phosphine-based ligands and found that 4-heptyl substituted bioxazoline (**L40**, BiOx) was the best ligand for the asymmetric transformation.^48^ An array of 2,2-diarylethylamines were afforded with high enantioselectivities and moderate to good yields.

### 4.2 Enantioselective arylation of benzyl C–H bonds

**Scheme 28:**
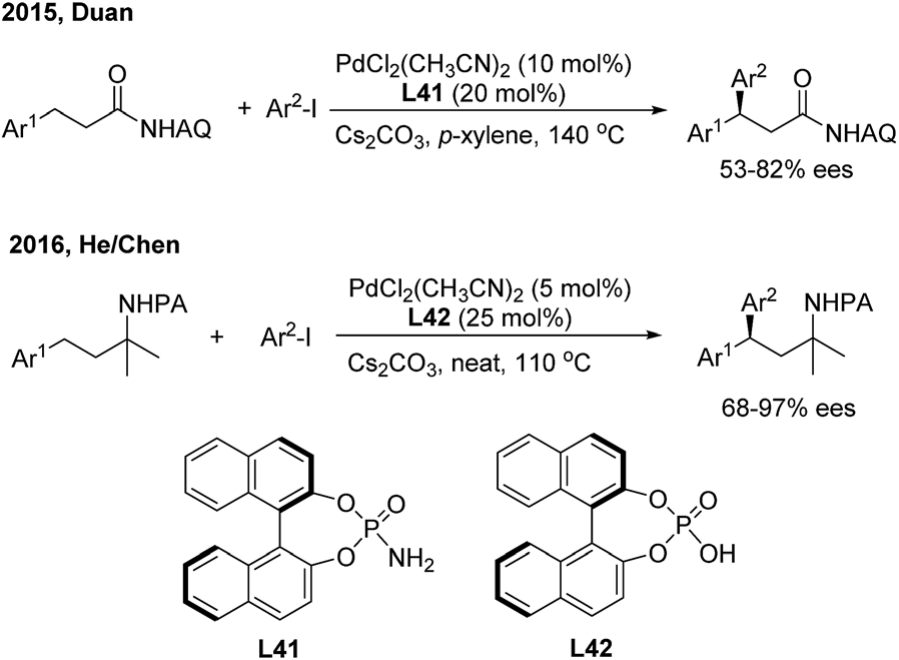
Pd-catalyzed and phosphate-mediated enantioselective benzylic C–H arylation reactions.

Transition metal-catalyzed asymmetric functionalizations of unreactive C–H bonds have been extensively investigated in recently years.^49^ The enantioselective arylation of benzylic C–H bonds enables direct access to optically active *gem*-diarylalkanes, wherein the precoordination of the metal catalyst with prochiral substrates in a bidentate or monodentate manner is usually demanded. In 2015, Duan^50^ firstly introduced a chiral phosphoric amide (**L41**) into the Pd(ii)-catalyzed direct β-arylation of aminoquinoline derived aliphatic amides with aryl iodides. An array of β,β-diaryl carboxylic acid derivatives were produced in moderate to good enantiomeric ratios (Scheme 28, top). One year later, He and Chen^51^ investigated the enantioselective γ-arylation of *N*-picolinic protected alkylamines with a combination of chiral phosphoric acid and Pd(ii) catalysts. In the end, both high yields and enantioselectivities were obtained using a substoichiometric amount of chiral phosphoric acid (**L42**) under solvent-free conditions (Scheme 28, bottom).

**Scheme 29:**
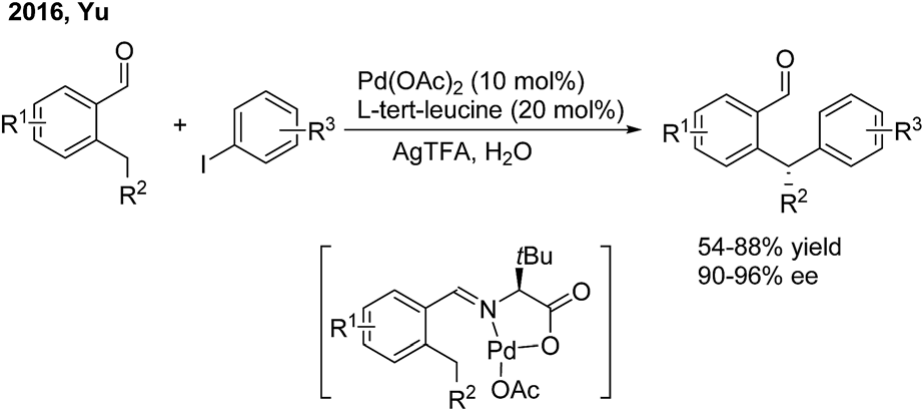
Pd-catalyzed and amino acid-mediated enantioselective benzylic C–H arylation of benzaldehydes.

In 2016, Yu employed chiral α-amino acids as transient directing groups in the enantioselective benzylic C(sp^3^)–H arylation of benzaldehydes *via* the precoordination of Pd(ii) with the *in situ* generated imine intermediate^52^ (Scheme 29). In the presence of 20 mol% l-*tert*-leucine, 10 mol% Pd(OAc)_2_ and 3 equiv. H_2_O, *o*-alkyl benzaldehydes reacted with a wide range of aryl iodides to produce 1,1-diaryl alkanes in moderate yields with high enantiomeric ratios.

**Scheme 30:**
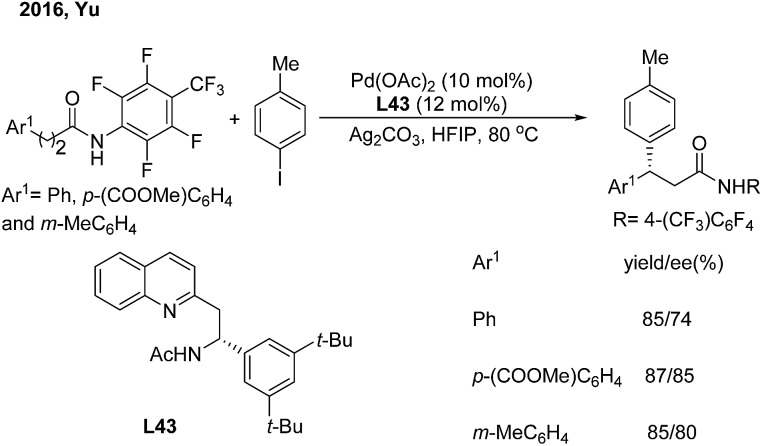
Pd-catalyzed enantioselective β-arylation of aliphatic amides induced by chiral acetyl-protected aminoethyl quinoline ligands.

Soon afterwards, the same group employed chiral acetyl-protected aminoethyl quinoline (**L43**) ligands in the Pd(ii)-catalyzed monodentate auxiliary directed C(sp^3^)–H arylation of aliphatic amides.^53^ This strategy enables the enantioselective construction of chiral 3,3-diaryl amides on subjecting 3-aryl propenamides to the catalytic system (Scheme 30).

**Scheme 31:**
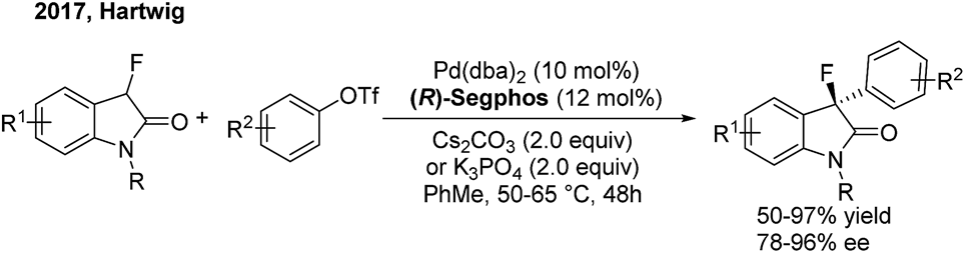
Pd-catalyzed enantioselective α-arylation of α-fluorooxindoles.

Although transition-metal catalysed asymmetric α-arylation of carbonyl compounds has been widely reported, the use of this method for the construction of *gem*-diarylalkanes has rarely been studied. In 2009, Buchwald^54^ disclosed a highly enantioselective Pd-catalyzed intermolecular C–C coupling of oxindoles and arylbromides using an axially chiral P-stereogenic ligand. Enantioenriched oxindoles containing a *gem*-diaryl quaternary center were afforded with 95–99% ee. Recently, Hartwig^55^ reported a palladium-catalyzed enantioselective α-arylation of α-fluorooxindoles with aryl triflates, using (*R*)-segphos as a chiral ligand. Enantioenriched 3-aryl-3-fluorooxindoles including a chiral quaternary center were obtained in high yields with excellent enantioselectivities (Scheme 31).

### 4.3 Enantioselective arylation of benzyl carbene precursors

**Scheme 32:**
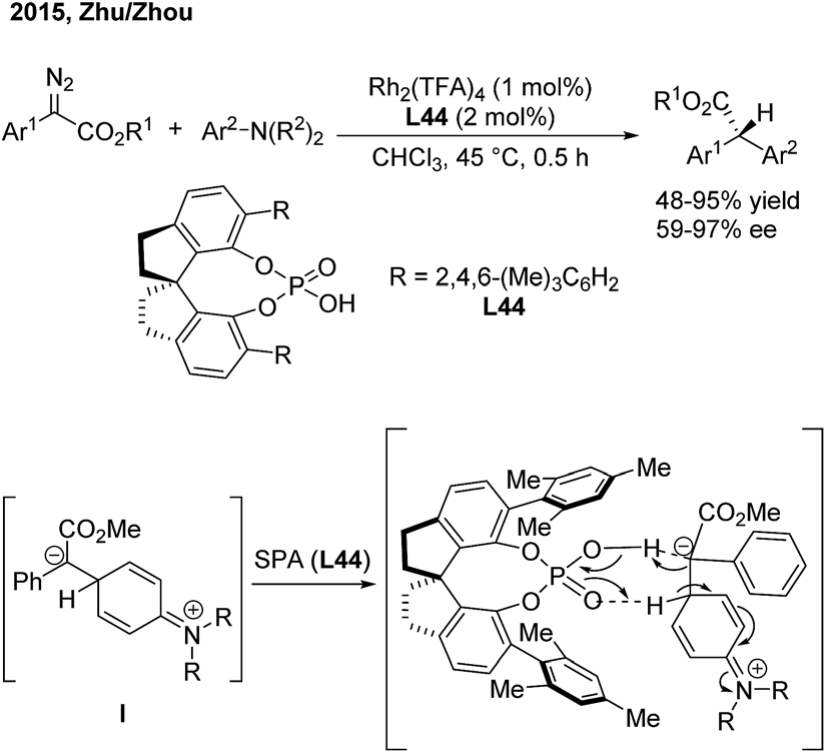
Catalytic asymmetric arylation of α-aryl-α-diazoacetates with aniline derivatives.

In 2015, Zhu and Zhou^56^ reported an enantioselective arylation of α-aryl-α-diazoacetates with anilines catalysed by dirhodium(ii) trifluoroacetate and a chiral spiro phosphoric acid (SPA) (Scheme 32). Chiral α-diaryl acetates were produced in good yields (up to 95%) and high enantioselectivities (up to 97% ee). A step-wise reaction mechanism was proposed based on deuterium-labeling experiments. The Rh_2_(TFA)_4_ catalyst is responsible for the generation of the zwitterion (**I**). The 1,2-proton shift occurs *via* a proton shuttle model, which is mediated and stereochemically controlled by the chiral SPA (**L44**).

## 5 Asymmetric aryl cross-coupling across C=C bonds

**Scheme 33:**
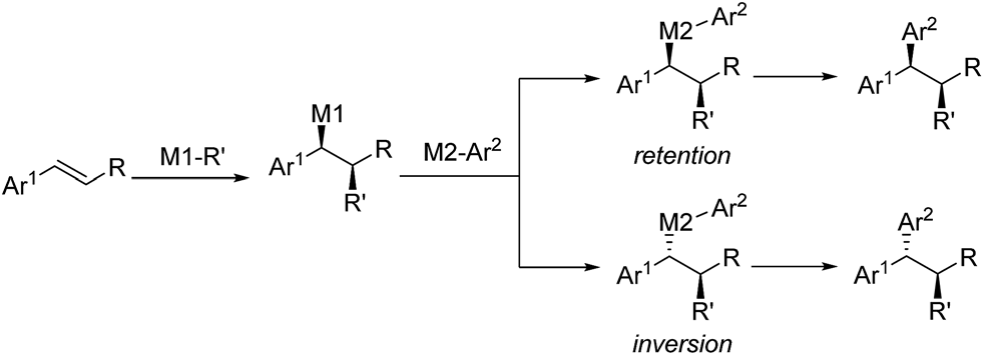
The conceptual strategy for the three-component cross-couplings.

Inspired by the efficiency of direct aryl-benzyl coupling, transition metal-catalyzed three-component cross-coupling reactions of olefins have been developed as an important and complementary method in the construction of *gem*-diaryl moieties. The conceptual strategy of this method involves the enantioselective formation from the styrene and stereospecific coupling of metal bound benzyl intermediates. These species are either nucleophilic or electrophilic depending on the nature of the initiator (M1-R′) (Scheme 33). In this regard, initiators include *in situ* generated Pd–H, Cu–H and Cu-Bpin and some electrophilic radicals (*i.e.* CF_3_ or amino radicals).

### 5.1 The net hydroarylation of styrene derivatives

**Scheme 34:**
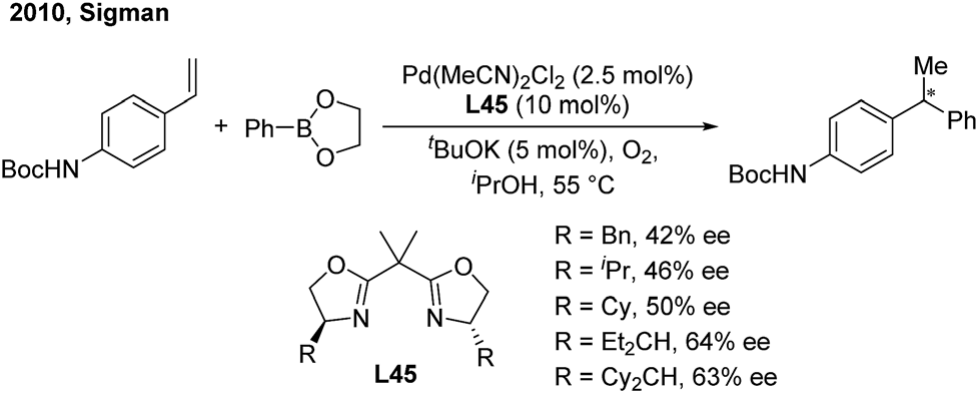
Pd-catalyzed hydroarylation of styrenes using bisoxazoline ligands.

In 2010, the Sigman group initially studied the palladium-catalyzed asymmetric hydroarylation of styrenes with arylboron esters in the presence of an i-PrOH solvent and in an O_2_ atmosphere^57^ (Scheme 34). Through investigating chiral NHC and bisoxazoline ligands, they found that bisoxazoline ligands (**L45**) could give the best enantioselective induction (up to 64%).

**Scheme 35:**
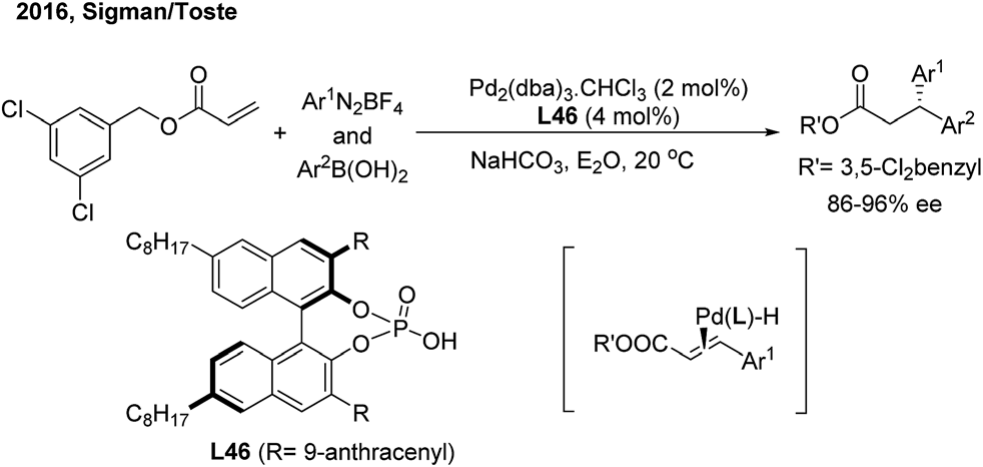
Pd-catalyzed enantioselective three-component cross-coupling of benzyl acrylates, aryldiazonium salts and arylboronic acids.

In 2016, Sigman and Toste developed an elegant enantioselective 1,1-diarylation method *via* double aryl cross-coupling to acrylates.^58^ They introduced the chiral anion phase transfer strategy into this diarylation transformation. Catalyzed by chiral phosphoric acid **L46** and Pd_2_(dba)_3_, optically active 3,3-diaryl esters with a high enantioselectivity were produced (Scheme 35). The process possibly involves a stereospecific hydroarylation of a chiral benzyl cinnamate-associated Pd(ii)–H complex intermediate.

**Scheme 36:**
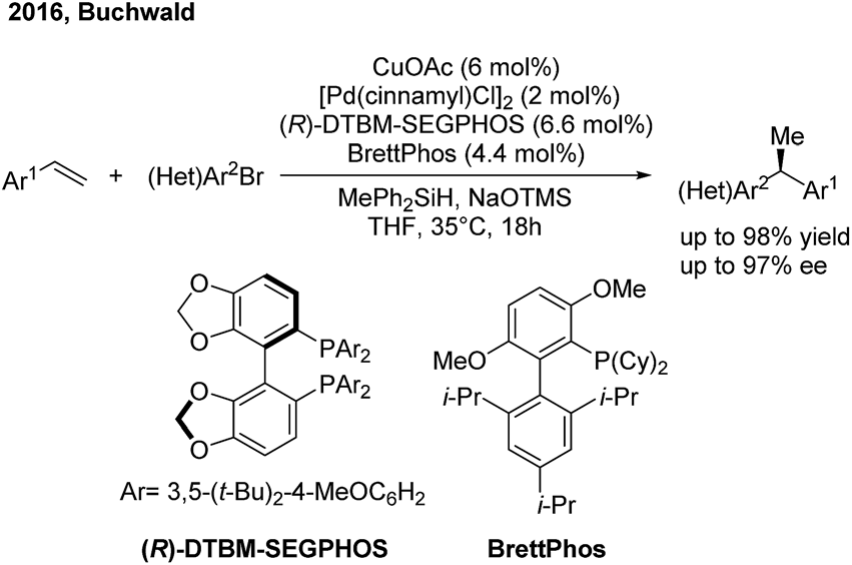
The cooperative Cu/Pd-catalyzed enantioselective hydroarylation of styrenes.

Recently the Buchwald group developed an alternative strategy to realize highly enantioselective hydroarylation of styrenes through CuH/Pd(0) cooperative catalysis^59^ (Scheme 36). In the presence of a chiral copper and achiral palladium catalyst, the three-component cross-coupling of styrenes, arylbromides and MePh_2_SiH proceeded smoothly to produce enantioenriched 1,1-diarylethanes in good yields with good to excellent enantioselectivities.

### 5.2 Borylarylation of styrene derivatives

**Scheme 37:**
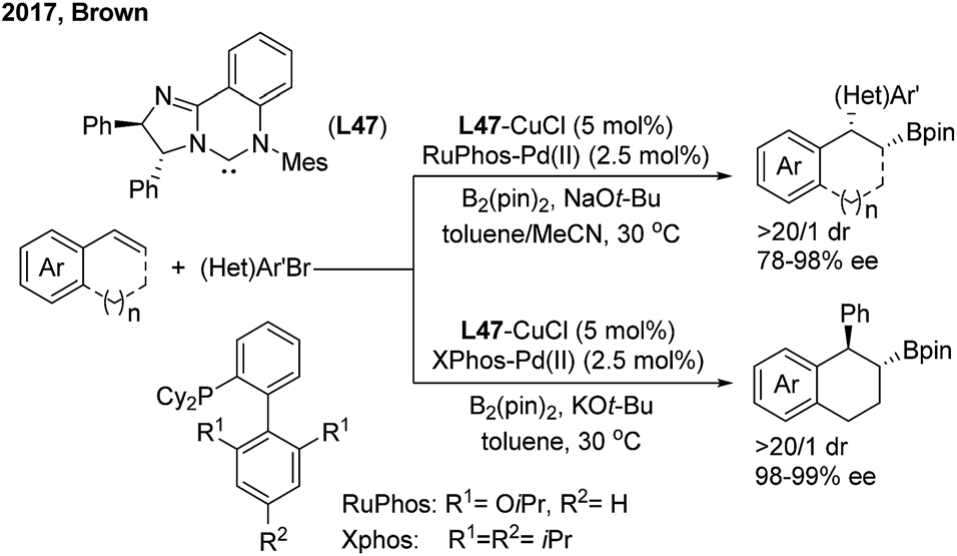
The cooperative Cu/Pd-catalyzed enantioselective 1,2-arylboration of vinylarenes using chiral NHC–carbene ligands.

The Cu/Pd cooperatively catalysed enantioselective 1,2-arylboration of styrenes was also demonstrated by the Brown^60^ and Liao^61^ groups independently. Brown found that the chiral NHC–carbene ligand (**L47**) was compatible with a variety of 1,2-bisubstituted alkyenylarene substrates with excellent diastereo- and enantioselectivities. The *syn*/*trans* selectivity of the arylboration addition of 1,2-dihydronaphthalene was facilely switched by changing the achiral ligands on the Pd(ii)-complex (Scheme 37).

**Scheme 38:**
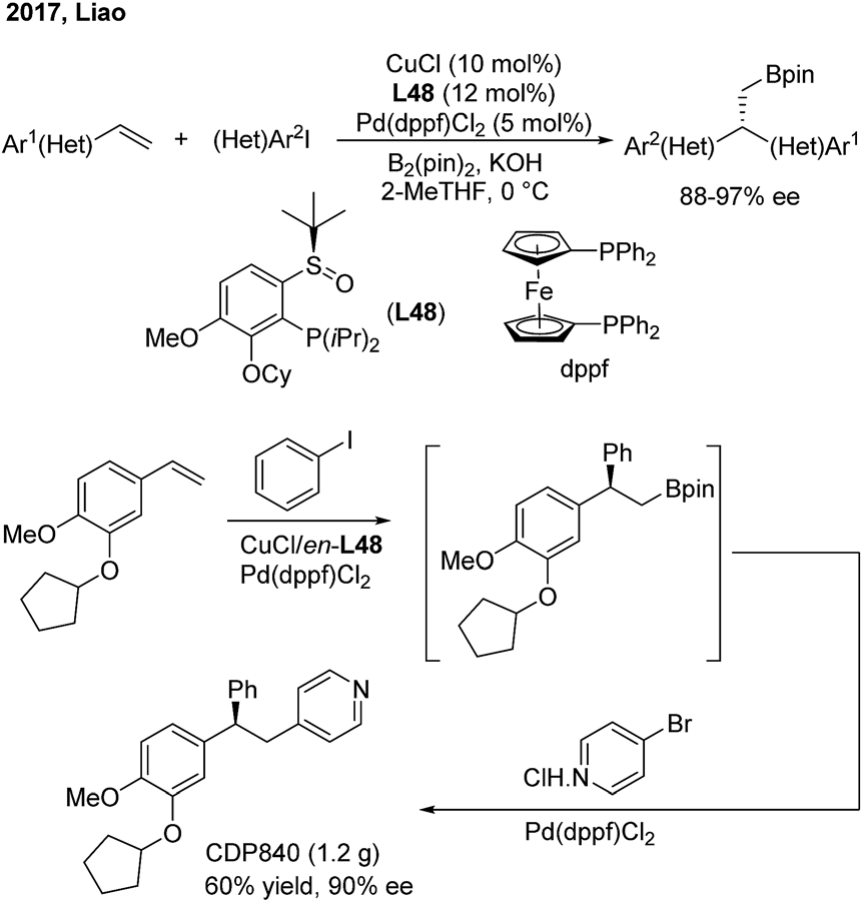
Chiral triarylethane synthesis through the enantioselective arylboration of styrenes.

Liao and co-workers utilized a chiral sulfoxide–phosphine ligand (**L48**) to promote the Cu/Pd-catalyzed enantioselective arylboration of terminal vinylarenes with aryl iodides under mild conditions (Scheme 38). The method was particularly effective for the synthesis of chiral 2,2′-heteroaryl-aryl-ethylborates from either heteroaryl alkenes or heteroaryl iodides. Furthermore, the author merged this transformation and Suzuki–Miyaura coupling into a streamlined procedure for the modular synthesis of a series of important 1,1,2-triarylethane molecules, including CDP840.

### 5.3 Trifluoromethyl and aminoarylation of styrene derivatives

**Scheme 39:**
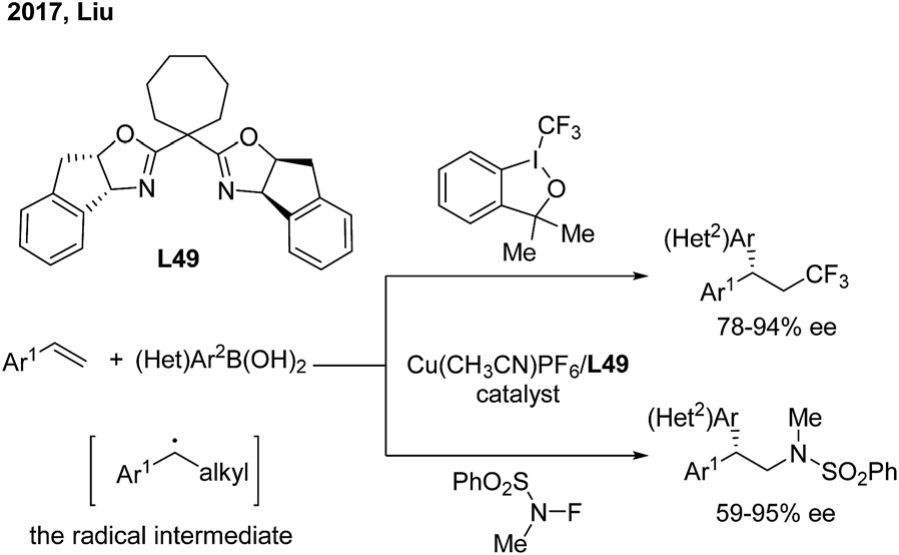
Cu/BOX-catalyzed enantioselective trifluoromethyl and aminoarylation of styrenes.

Recently, Liu and coworkers developed a novel copper catalysis strategy to construct a *gem*-diarylmethine stereogenic center *via* enantioselective arylation of a secondary benzyl radical intermediate.^62^ In the presence of a Cu(i)/**L49** catalyst, the enantioselective trifluoromethyl and aminoarylation of styrenes proceeded smoothly and afforded *gem*-diarylethane derivatives in moderate to high yields and with good ees (Scheme 39).

## 6 Conclusion and perspective

In this review, a large number of TMCAAr reactions, which target the construction of chiral *gem*-diaryl tertiary or quaternary stereogenic centers, have been described. These reactions are versatile methods to site-selectively and stereochemically couple prochiral or racemic starting materials with various aryl reagents (almost always aryl metals or halides) to provide nonracemic *gem*-diarylalkane compounds. Due to distinguishing features including the wide range of substrate scope, good functional group tolerance and the use of easily accessible substrates, the related methodologies have received increasing interest from synthetic and pharmaceutical chemists, aiding the latter in synthesising medicinal molecules in a highly efficient manner.

Predictably, the development of strategies that transform commercially available feedstocks to highly valuable *gem*-diaryl molecules has recently been highlighted and will be the focus of continuous research. The present methods, including hydro- or borylarylation, direct benzyl C–H bond arylation and so on, need improvement of the efficiency (*i.e.* enantioselectivities and catalyst loadings) and broadening of the substrate scope, and their use in the construction of quaternary carbon stereogenic centres remains challenging.

## Conflicts of interest

There are no conflicts to declare.

## References

[cit1] Ameen D., Snape T. J. (2013). Med. Chem. Commun..

[cit2] Hayashi T., Yamasaki K. (2003). Chem. Rev..

[cit3] Paquin J.-F., Defieber C., Stephenson C. R. J., Carreira E. M. (2005). J. Am. Chem. Soc..

[cit4] Itoh T., Mase T., Nishikata T., Iyama T., Tachikawa H., Kobayashi Y., Yamamoto Y., Miyaura N. (2006). Tetrahedron.

[cit5] Nishikata T., Yamamoto Y., Gridnev I. D., Miyaura N. (2005). Organometallics.

[cit6] Chen G., Tokunaga N., Hayashi T. (2005). Org. Lett..

[cit7] Chen G., Xing J., Cao P., Liao J. (2012). Tetrahedron.

[cit8] Ming J., Hayashi T. (2016). Org. Lett..

[cit9] Duan H. F., Xie J. H., Qiao X. C., Wang L. X., Zhou Q. L. (2008). Angew. Chem., Int. Ed..

[cit10] Wang J., Wang M., Cao P., Jiang L., Chen G., Liao J. (2014). Angew. Chem., Int. Ed..

[cit11] Lee A., Kim H. (2015). J. Am. Chem. Soc..

[cit12] Takatsu K., Shintani R., Hayashi T. (2011). Angew. Chem., Int. Ed..

[cit13] Wu C., Yue G., Nielsen C. D.-T., Xu K., Hirao H., Zhou J. (2016). J. Am. Chem. Soc..

[cit14] Duursma A., Hoen R., Schuppan J., Hulst R., Minnaard A. J., Feringa B. L. (2003). Org. Lett..

[cit15] Jumde V. R., Iuliano A. (2013). Adv. Synth. Catal..

[cit16] Wang Z. Q., Feng C. G., Zhang S. S., Xu M. H., Lin G. Q. (2010). Angew. Chem., Int. Ed..

[cit17] Huang K. C., Gopula B., Kuo T. S., Chiang C. W., Wu P. Y., Henschke J. P., Wu H. L. (2013). Org. Lett..

[cit18] Li R., Wen Z., Wu N. (2016). Org. Biomol. Chem..

[cit19] Lang F., Chen G., Li L., Xing J., Han F., Cun L., Liao J. (2011). Chem.–Eur. J..

[cit20] Bao X., Cao Y.-X., Chu W.-D., Qu H., Du J.-Y., Zhao X.-H., Ma X.-Y., Wang C.-T., Fan C.-A. (2013). Angew. Chem., Int. Ed..

[cit21] Xue F., Wang D., Li X., Wan B. (2012). J. Org. Chem..

[cit22] Sieber J. D., Rivalti D., Herbage M. A., Masters J. T., Fandrick K. R., Fandrick D. R., Haddad N., Lee H., Yee N. K., Gupton B. F., Senanayakea C. H. (2016). Org. Chem. Front..

[cit23] He Q., Xie F., Fu G., Quan M., Shen C., Yang G., Gridnev I. D., Zhang W. (2015). Org. Lett..

[cit24] Mauleoón P., Carretero J. C. (2004). Org. Lett..

[cit25] Nishimura T., Takiguchi Y., Hayashi T. (2012). J. Am. Chem. Soc..

[cit26] Yu Y.-N., Xu M.-H. (2014). Acta Chim. Sin..

[cit27] Thornbury R. T., Saini V., Fernandes T. de A., Santiago C. B., Talbot E. P. A., Sigman M. S., McKenna J. M., Toste F. D. (2017). Chem. Sci..

[cit28] Cahard E., Male H. P. J., Tissot M., Gaunt M. J. (2015). J. Am. Chem. Soc..

[cit29] Dosa P. I., Fu G. C. (1998). J. Am. Chem. Soc..

[cit30] García C., Walsh P. J. (2003). Org. Lett..

[cit31] Chen C.-A., Wu K.-H., Gau H.-M. (2007). Angew. Chem., Int.Ed..

[cit32] Korenaga T., Ko A., Uotani K., Tanaka Y., Sakai T. (2011). Angew. Chem., Int. Ed..

[cit33] Duan H. F., Xie J. H., Qiao X. C., Wang L. X., Zhou Q. L. (2008). Angew. Chem., Int. Ed..

[cit34] Martina S. L., Jagt R. B., de Vries J. G., Feringa B. L., Minnaard A. J. (2006). Chem. Commun..

[cit35] Shintani R., Inoue M., Hayashi T. (2006). Angew. Chem., Int. Ed..

[cit36] Lai H., Huang Z., Wu Q., Qin Y. (2008). J. Org. Chem..

[cit37] Tomita D., Yamatsugu K., Kanai M., Shibasaki M. (2009). J. Am. Chem. Soc..

[cit38] Yamamoto Y., Yohda M., Shirai T., Ito H., Miyaura N. (2012). Chem.–Asian J..

[cit39] Shintani R., Takeda M., Tsuji T., Hayashi T. (2010). J. Am. Chem. Soc..

[cit40] Wang H., Jiang T., Xu M. H. (2013). J. Am. Chem. Soc..

[cit41] Yang G., Zhang W. (2013). Angew. Chem., Int. Ed..

[cit42] Jiang C., Lu Y., Hayashi T. (2014). Angew. Chem., Int. Ed..

[cit43] Alexakis A., Hajjaji S. E., Polet D., Rathgeb X. (2007). Org. Lett..

[cit44] Tian H., Zhang P., Peng F., Yang H., Fu H. (2017). Org. Lett..

[cit45] Selim K. B., Yamada K., Tomioka K. (2008). Chem. Commun..

[cit46] Do H.-Q., Chandrashekar E. R. R., Fu G. C. (2013). J. Am. Chem. Soc..

[cit47] Poremba K. E., Kadunce N. T., Suzuki N., Cherney A. H., Reisman S. E. (2017). J. Am. Chem. Soc..

[cit48] Woods B. P., Orlandi M., Huang C. Y., Sigman M. S., Doyle A. G. (2017). J. Am. Chem. Soc..

[cit49] Newton C. G., Wang S.-G., Oliveira C. C., Cramer N. (2017). Chem. Rev..

[cit50] Yan S.-B., Zhang S., Duan W.-L. (2015). Org. Lett..

[cit51] Wang H., Tong H.-R., He G., Chen G. (2016). Angew. Chem., Int. Ed..

[cit52] Zhang F.-L., Hong K., Li T.-J., Park H., Yu J.-Q. (2016). Science.

[cit53] Chen G., Gong W., Zhuang Z., Andrä M. S., Chen Y.-Q., Hong X., Yang Y.-F., Liu T., Houk K. N., Yu J.-Q. (2016). Science.

[cit54] Taylor A. M., Altman R. A., Buchwald S. L. (2009). J. Am. Chem. Soc..

[cit55] Jin Y.-S., Chen M., Ge S.-Z., Hartwig J. F. (2017). Org. Lett..

[cit56] Xu B., Li M. L., Zuo X. D., Zhu S. F., Zhou Q. L. (2015). J. Am. Chem. Soc..

[cit57] Podhajsky S. M., Iwai Y., Cook-Sneathen A., Sigman M. S. (2011). Tetrahedron.

[cit58] Yamamoto E., Hilton M. J., Orlandi M., Saini V., Toste F. D., Sigman M. S. (2016). J. Am. Chem. Soc..

[cit59] Friis S. D., Pirnot M. T., Buchwald S. L. (2016). J. Am. Chem. Soc..

[cit60] Logan K. M., Brown M. K. (2017). Angew. Chem., Int. Ed..

[cit61] Chen B., Cao P., Yin X., Liao Y., Jiang L., Ye J., Wang M., Liao J. (2017). ACS Catal..

[cit62] Wu L., Wang F., Wan X., Chen D. W., Chen P., Liu G. (2017). J. Am. Chem. Soc..

